# Community-based perinatal mental health peer support: a realist review

**DOI:** 10.1186/s12884-023-05843-8

**Published:** 2023-08-09

**Authors:** Jenny McLeish, Susan Ayers, Christine McCourt

**Affiliations:** Centre for Maternal and Child Health Research, School of Health Sciences, City, University of London, 1 Myddelton Street, London, EC1R 1UW UK

**Keywords:** Peer support, Mental health, Antenatal, Postnatal, Community-based, Realist, Context, Mechanism

## Abstract

**Background:**

Peer support has been suggested as an alternative or complement to professional support for mothers with perinatal mental health difficulties. The aim of this realist review was to synthesise the evidence on perinatal mental health peer support programmes outside mental health services, to understand what is it about community-based perinatal mental health peer support that works, for whom, in what circumstances, in what respects, and why.

**Methods:**

Applying realist methodology, an initial theoretical model was tested against evidence from empirical studies. 29 empirical studies were included, covering 22 antenatal and postnatal mental health interventions that offered one-to-one or group peer support, in person or by telephone. Data extraction identified the configurations of contexts (C), mechanisms (M) and outcomes (O) relevant to mothers’ use of peer support and to the positive and negative effects of using peer support.

**Results:**

13 C-M-O configurations explained take-up of peer support. These were based on mothers’ perceptions that peer support would offer empathetic understanding and non-judgemental acceptance outside their social circle; their relationships with primary health professionals; their cultural background and perspectives on mental health; their desire for professional support; overcoming practical barriers; the format of the support; and the use of volunteers. A further 13 C-M-O configurations explained positive impact on mothers. These were based on receiving empathetic listening, acceptance, affirmation and normalisation; peers sharing ideas about self-care, coping, and services; peers using therapeutic techniques; the opportunity to give support to others; meaningful social relationships with volunteers and other mothers; and other benefits of attending a group. There were 8 C-M-O configurations explaining negative impact. These were based on lack of validation; self-criticism from downward and upward social comparison; a culture of negativity; peers being judgemental or directive; not feeling heard; peer support as a stressful social relationship; and distress at endings.

**Conclusions:**

Peer support works in complex ways that are affected by personal and social contexts. Providers, commissioners and evaluators can use this review to understand and maximise the valuable benefits of peer support, to minimise potential risks, and to devise ways of reaching mothers who do not currently engage with it.

**Supplementary Information:**

The online version contains supplementary material available at 10.1186/s12884-023-05843-8.

## Background

Mental health difficulties in the perinatal period can affect both the mother’s wellbeing and her baby’s physical, psychological, mental, emotional and behavioural development [1–5]. Peer support - that is, organised social support from another woman who has or has had perinatal mental health difficulties - is one among a range of psychosocial interventions that have been tested to help women with perinatal mental health difficulties [6, 7]. Perinatal mental health peer support in the United Kingdom (UK) includes some formal roles in inpatient settings and community mental health teams [8], and peer support offered outside the National Health Service by an increasing number of third sector organisations ranging in size from national charities to informal local groups [9, 10]. The Royal College of Psychiatrists has acknowledged the important role of peer support provided by the third sector as an alternative to professional support for women with mild perinatal mental health difficulties, or alongside or after professional support for those with more serious perinatal mental health difficulties [9]. Community-based third sector programmes in the UK usually offer their perinatal peer support in groups or one-to-one from trained peer supporters (mainly unpaid volunteers), but many operate without an evaluated delivery model [11, 12].

In existing reviews, perinatal peer support from women with their own experience of mental health difficulties has been analysed (a) with different interventions such as therapy groups and peer education [7, 13–19], and/or (b) with other forms of social support, usually from mothers who do not have experience of perinatal mental health difficulties [7, 20–22]. When perinatal mental health peer support was separated out from other interventions or forms of social support, these systematic reviews found no evidence that peer support had an impact on anxiety symptoms, and some evidence of a moderate impact on depression symptoms (but not diagnosis) at endpoint [15]. Qualitative synthesis of group interventions suggested that perinatal peer support could help mothers overcome isolation, gain hope of recovery, gain confidence by leaving the house, realise that others have similar experiences, and learn to recognise the symptoms of postnatal depression, but there were also some negative effects: mothers feeling over-reliant on the group, and having anxiety reinforced by social comparison with another participant who was not getting better [18]. Otherwise there was little attention given by these reviews to the potential negative aspects of peer support, reflecting the lack of attention to negative impacts in the literature. It has been noted that women who participate in perinatal mental health peer support groups are more likely to be socially advantaged [23], and that mental health peer support in general may be less appealing to people from minoritised communities who may prefer to affiliate with others based on other identifies and experiences [24].

Many researchers have used middle range theories (explanatory theories which are capable of being a ground for prediction and tested with data) [25] to explain how mental health peer support can create psychological outcomes [13, 23, 26–39]. The middle range theories that have been applied include social comparison theory [40], overcoming stigma through reflected appraisal [41], peer support groups as normative narrative communities [33], experiential knowledge and expertise [42], multi-dimensional social support [43], stress-buffering through coping assistance [44], helper-therapy [45], and attachment theory [46]. These theories provide potential explanations for the causal mechanisms of peer support, but have not yet been integrated into an analysis of the personal and social contexts in which they may or may not cause psychological outcomes for different people.

In order to assist the future development of safe and effective perinatal mental health peer support in the third sector, there is a need to develop this limited evidence base. In particular it is important to understand why some women take up peer support while others do not, and why and how peer support helps some women but not others, so that peer support programmes can maximise the benefits and minimise the risks. This review therefore aims to synthesise the evidence on perinatal mental health peer support programmes outside mental health services, to understand what is it about community-based perinatal mental health peer support that works, for whom, in what circumstances, in what respects, and why.

## Methods

Realist review is a methodology for reviewing literature that aims to *explain* social programmes rather than assessing their average effects, based on an understanding of the social world as an open system where latent causal mechanisms (M) are activated by particular contexts (C) and interact with one another in complex ways to generate new phenomena, including positive and negative outcomes (O) [47–49]. A social programme introduces new resources with the intention of addressing a problem, and the mechanisms of change are the reasoning and reactions of participants in response to the resources provided by the programme [50, 51].

Programmes are understood to work in different ways in different circumstances and for different people because mechanisms are emergent phenomena affected by contexts, which could be macro-level factors such as socio-cultural values, meso-level factors such as the setting for the programme, or micro-level factors such as the personal characteristics of those involved [52, 53]. Synthesising information from different studies enables a theoretical model for that type of programme to be created, based on context-mechanism-outcome (C-M-O) configurations which are also known as ‘programme theories’.

This realist review followed the steps proposed by the RAMESES guidelines [54], in which a hypothesised initial theoretical model is created based on exploratory searching, and is then tested against empirical data gathered in the second stage of searching, to create a final theoretical model of how and why the programme works. The question for the review was: ‘What is it (M) about community-based perinatal mental health peer support that works (O), for whom (C), in what circumstances (C), in what respects (O), and why (M)?” The review began with exploratory searching for middle range theories relevant to peer support mechanisms and outcomes, and for studies of maternal mental health relevant to contexts. This included searches of Medline, Google Scholar, and policy and practice websites (https://www.england.nhs.uk, https://www.gov.uk, https://fingertips.phe.org.uk/profile-group/mental-health/profile/perinatal-mental-health, https://www.rcpsych.ac.uk/, https://www.nice.org.uk, https://maternalmentalhealthalliance.org). Women with lived experience of perinatal mental health difficulties, third sector programme leads, and local authority commissioners were asked for their views of how perinatal mental health peer support ‘works’, in informal discussions at eight conferences, a programme of perinatal mental health masterclasses, and a project advisory group for a new community-based perinatal mental health peer support programme.

The results of exploratory searching were used to construct an initial theoretical model that addressed take-up of peer support, positive impact on mothers of using peer support, and negative impact on mothers of using peer support. The evidence sources used to inform the initial theoretical model are shown in Additional File 1 for contexts and Additional File 2 for mechanisms and outcomes. The retroductive realist question ‘what must be true for this to be the case?’ was used to theorise how partial C-M-O configurations could be developed more fully, working backwards from effects to the conditions that would be necessary for those effects to be produced.

The second stage was a search for empirical studies of perinatal mental health peer support interventions, which was purposively rather than methodologically driven [53], and included qualitative, quantitative and mixed methods studies, as well as literature generated by third sector peer support programmes in England. Searches were carried out in four databases, without date restrictions: Cumulative Index to Nursing and Allied Health Literature, PubMed, Scopus and PsychInfo, with the last search conducted in March 2020, using these search terms:

Perinatal OR Pregnan* OR Antenatal* OR Postnatal* OR Postpartum OR Maternal OR Parent* OR Mother*.

AND Mental health OR Mental illness OR Emotional* OR Wellbeing* OR Depress* OR Anxiety OR Anxious OR PND OR OCD OR Psychosis.

AND Peer support* OR Peer work* OR Volunteer* OR Peer* OR Community run organi* OR self-help OR self help OR support group OR consumer-provider OR consumer.

A search was also carried out in the British Library E-theses Online service. Backward citations in included papers were searched. Eleven local and national third sector organisations providing community-based perinatal mental health peer support were contacted by email to request project reports, evaluations or other literature, and three responded.

The inclusion and exclusion criteria for the empirical studies are shown in Table 1.

**Table 1:** Selection criteria for empirical studies

	Inclusion criteria	Exclusion criteria
**Type of study**	Empirical study of participant experiences, outcomes or process.	Review article
**Methodology**	Any	
**Population**	Women experiencing any type and level of perinatal mental health difficulties, diagnosed or self-identified.	• Women outside the period of pregnancy and the first two years after birth. • Women who did not identify themselves as having any perinatal mental health difficulty.
**Intervention**	Interventions offering peer support for perinatal mental health difficulties, face-to-face or by telephone, including: • One-to-one peer support from trained peers (with personal experience of perinatal mental health difficulties). • Peer support groups facilitated by peers or non-peers.	• Interventions combining psychological therapy and peer support. • Groups facilitated by a mental health professional. • Peer support interventions not primarily focused on perinatal mental health difficulties. • Interventions aimed at preventing perinatal mental health difficulties. • Interventions primarily offering peer education. • Interventions based on internet chat forums.
**Setting**	• Interventions based in the community. • Interventions in any country.	• Peer support offered as part of a mental health service. • Peer support in in-patient settings.

In realist review, quality assessment is not applied to whole documents as a basis for inclusion but to each piece of information extracted, applying the considerations of relevance and rigour (Wong et al., 2013). Each included document was read closely and critically as part of the data extraction process. Data relevant to contexts, mechanisms and proximate outcomes were judged according to how reliably the specific piece of information was generated, mindful of the strengths and weaknesses of the whole study which was guided by the appropriate quality criteria from the Mixed Methods Appraisal Tool (MMAT) [55].

Texts of the included documents were entered into NVIVO software. Nodes were created for the draft programme theories (C-M-O configurations) from the initial theoretical model. Documents were coded in an iterative process as described by Dalkin et al. [56]. Deductive coding was based on the original draft programme theory nodes. New nodes were created for inductive coding where the analysis suggested a new C-M-O configuration not anticipated in the initial theoretical model. Further new nodes were created to represent partial programme theories, for example, a context and mechanism but no outcome.

Each programme theory in the initial theoretical model was then tested against the evidence drawn from the included studies, using the results of the C-M-O analysis. Where the C-M-O analysis indicated a new programme theory not anticipated in the initial theoretical model, or a refinement to an existing theory, this was added to the model. The final theoretical model was thus a combination of those parts of the initial theoretical model that were supported by the C-M-O analysis, and new programme theories identified through the C-M-O analysis. Different fonts were used in the final theoretical model to indicate theories that were present in both initial and final models (normal font), theories that were in the initial model but not in the final model because no evidence was found (strikethrough font), and theories that were not in the initial model but were in the final model (capitalised font).

## Results

**Fig. 1:**
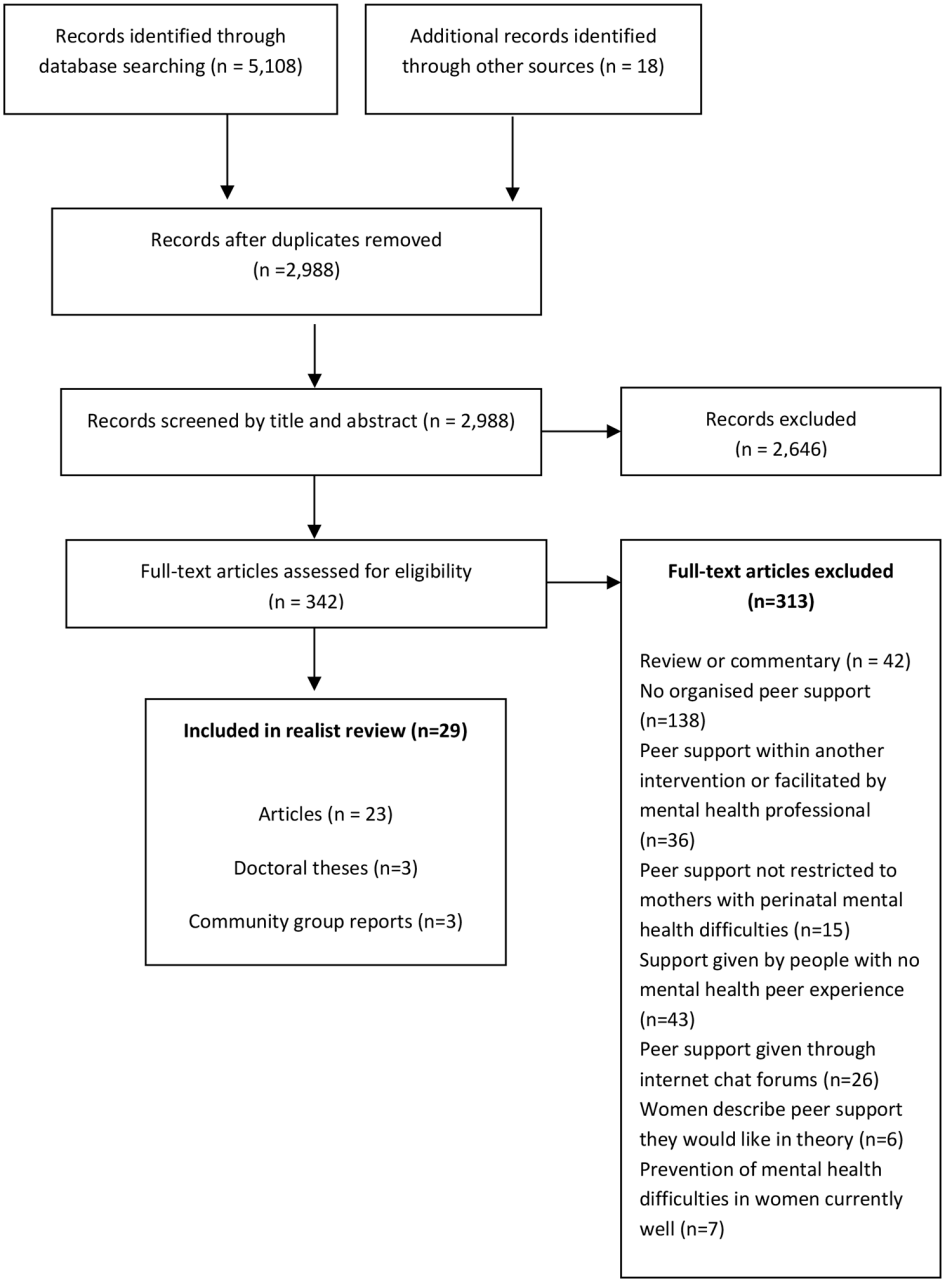
Results of the search for empirical studies on perinatal mental health peer support

The search for empirical studies identified 5,126 documents, of which 29 were included in the realist review – 23 peer-reviewed articles [57–79], three doctoral theses [80–82], and three reports from community groups [83–85]. These comprised eight qualitative studies, seven randomised controlled trials, five non-randomised quantitative studies, three quantitative descriptive studies, two mixed methods (randomised controlled trial and qualitative) studies, and four descriptions of process. The search results are shown in Fig. 1 and the quality assessment is shown in Additional File 3. These 29 documents described a total of 22 perinatal mental health peer support interventions, each of which was given an intervention number (#), shown in Table 2. Details of the interventions are shown in Additional File 4.

**Table 2:** Included documents grouped by intervention number

Intervention #	Author (date), [article reference],
1	Anderson (2013) [57]
2	Cust (2016) [58] Carter et al. (2018) [59]
3	Carter et al. (2019) [60] Cust and Carter (2018) [61]
4	Chen et al. (2000) [62]
5	Dennis (2003) [63]
6	Dennis et al. (2009) [64] Dennis (2010) [65] Dennis (2013) [66] Dennis (2014) [67]
7	Duskin (2005) [80]
8	Eastwood et al. (1995) [68]
9	Field et al. (2013a) [69]
10	Field et al. (2013b) [70]
11	Gjerdingen et al. (2013) [71]
12	Letourneau et al. (2015) [72] Letourneau et al. (2016) [73]
13	Ludwick (2017) [81]
14	Maley (2002) [74]
15	Montgomery et al. (2012) [75]
16	Pitts (1999) [76]
17	Prevatt et al. (2018) [77]
18	Sembi (2018) [82]
19	Shorey et al. (2019) [78] Shorey and Ng (2019) [79]
20	Acacia Family Support (2019) [83]
21	Fairbairn and Kitchener (2020) [84]
22	Lynch (2019) [85]

It was rare for evidence of a full C-M-O configuration to appear in an individual document. For the topic of mothers taking up peer support, there was abundant evidence for potential contexts but the mechanisms were not usually explicit, as the studies generally did not investigate why their participants had decided to use peer support, although some had obtained information about the reasons why other mothers had chosen *not* to use it. For the topics of how peer support may work positively or negatively, there was abundant evidence for mechanisms, sometimes explicitly linked to outcomes, while the contexts were often implied (or needed to be inferred through retroduction), and it was not possible to link measurable mental health outcomes to specific C-M-Os. The only evidence about meso-level contexts (such as programme setting and organisation) related to volunteer training and the local acceptability of telehealth. The theoretical model as presented therefore retains a level of hypothesis and scope for future exploration of full C-M-O linkages through realist evaluation.

### Summary of interventions

Three interventions offered one-to-one support in person from trained volunteers (interventions #2,3,20), one offered in-person support from a paid peer supporter (#21), and 11 offered in-person group support (#1,4,7–10,14–17,22). Six offered one-to-one telephone support from a trained peer volunteer (#5,6,11,12,18,19), with interventions #6, 12 and 18 being based on the approach piloted in #5. There was an unsuccessful attempt to deliver group telephone support in one intervention (#13) and this was also inspired by #5. Eight interventions took place in the USA (#1,7,9–11,13–15), eight in the UK (#2,3, 6,16,18,20–22), four in Canada (#5,6, 12,15), one in Taiwan (#4) and one in Singapore (#19).

Most interventions were for mothers with postnatal depression, which was either self-defined by the mother (#1,7,13–15), or assessed using a validated self-report instrument (#2,4–6,8,11,12,16,18); only three had an upper threshold for scores (#2,12,18). Three interventions were for antenatal depression and identified eligible mothers using the Whooley screening questions [86] (#3) or a structured clinical interview (#9,10). Two interventions were open to mothers with antenatal or postnatal depression or anxiety, self-defined (#20) or assessed using a validated self-report instrument (#21) and one was for any self-defined perinatal mental health difficulty (#22).

The length of interventions with a planned number of sessions ranged from four weeks to four months, but some gave no details about length or intensity (#1,7,11) and some allowed the one-to-one peer support to continue past the expected length if the volunteer and mother chose this (#5,6,19). The frequency was weekly in almost all interventions that had a fixed number of sessions, and monthly for one group (#14).

### Take-up and drop-out

There was evidence about take-up rates for eight interventions and about early drop-out for eleven interventions that had a planned length. There was considerable variation in the proportion of mothers who took up peer support. When support was offered directly, it was accepted by 37% of mothers in intervention #12 and 52% of mothers in intervention #21. Where mothers were invited to take part in a peer support trial, initial recruitment ranged from 38% (#18) to 72% (#6). There were unanticipated difficulties in recruiting mothers to receive peer support reported in interventions #8, 13, 14 and 18. Reported drop-out rates were generally low (e.g. #9,10) or none at all (e.g. #2,3). Half of participants dropped out of intervention 18, and there was an unexpected finding that slightly more participants dropped out from the intervention group who received peer support telephone calls, than from the control group.

### Summary of theories related to take-up, positive and negative impact

There were 13 C-M-O configurations relating to *take up* of perinatal mental health peer support, shown in Additional File 5. These programme theories were based on mothers’ expectations that peer support would offer empathetic understanding and non-judgemental acceptance outside their social circle (theories 1–3); mothers’ relationships with primary health professionals (theories 4 and 5); their cultural background and perspectives on mental health (theories 6–8); their desire for professional support (theory 9); overcoming barriers of time and money (theory 10); the format of the support (theories 11–12); and the use of volunteers (theory 13).

There were a further 13 C-M-O configurations explaining *positive impact* on mothers, shown in Additional File 6. These theories were based on receiving empathetic listening, acceptance, affirmation and normalisation (theories 14–17); peers sharing ideas about self-care, coping, and services (theory 18); peers using therapeutic techniques (theory 19); the opportunity to give support to others (theory 20); meaningful social relationships with volunteers and other mothers (theories 21–22); and other benefits of attending a group (theories 23–24).

There were also 8 C-M-O configurations explaining *negative impact* on mothers, shown in Additional File 7, although there was much less evidence for these. These theories were based on lack of validation (negative theory 1); self-criticism from downward and upward social comparison (negative theories 2–3); a culture of negativity (negative theory 4); peers being judgemental or directive (negative theory 5); not feeling heard (negative theory 6), peer support as a stressful social relationship (negative theory 7) and distress at endings (negative theory 8). There was no evidence for two further hypothesised negative C-M-Os, based on peers sharing unhelpful ideas and failure of social relationships.

## Discussion

Community-based peer support programmes are heterogeneous in the format of what they offer and their criteria for who can make use of their support, and mothers with perinatal health difficulties have diverse needs, experiences and ideas. By seeking out the differences as well as the commonalities, and linking contexts to mechanisms and outcomes, this realist review has generated a more complex picture of perinatal mental health peer support than portrayed in previous qualitative syntheses or theoretical models. Most of the theories that appeared in the final theoretical model had been hypothesised in the initial model, but there were some entirely new C-M-O configurations identified from the empirical studies and there were also new individual contexts and mechanisms added to some theories.

Community-based peer support is sometimes positioned as a more acceptable alternative to professional support for people who feel stigmatised by their mental health difficulties [87]. While there was evidence that this was true for some mothers who had disappointing previous experiences, feared being judged or believed that lived experience was a stronger basis for giving information than professional knowledge (theory 4), others had used peer support because they were unable to access the professional support that they wanted (theory 9), or by referral from a professional who they trusted and to whom they had disclosed their difficulties (theory 5). Just as some women may not access professional support because they do not understand their distress in terms of mental health difficulties that can be helped [88], it was a precondition of a mother taking up peer support that the mother herself *believed* it would be beneficial to talk about her problems (theory 7). It is the fundamental premise of mental health peer support that peers will understand each other’s situation empathically [34], but in order for a mother who wanted to talk about her feelings to choose peer support it was necessary firstly, that she wanted to talk to someone outside her social circle (theory 2) and secondly, that she *believed* that women with peer experiences would be empathetically understanding and non-judgmentally accepting (theories 1 and 3).

As predicted by work on peer support in many different contexts [89], across all interventions there was very strong evidence that, once mothers took up peer support, it was a means of overcoming shame and stigma through emotional and appraisal (esteem) support leading to emotion-focused and perception-focused coping [44]. Mothers experienced empathetic, non-judgemental understanding from peers, and they received positive feedback about their feelings and actions. This acceptance and affirmation enabled mothers to see themselves as worthy of acceptance and encouraged further self-disclosure [90].

Another strongly evidenced strand of peer support was hearing other women talk about their own perinatal mental health and parenting challenges, which normalised what mothers had previously believed to be a highly abnormal experience. This lateral social comparison [40] helped to overcome their sense of unique ‘failure’ by creating a new story about the meaning of their experiences - a normative narrative community [33], which enabled mothers to practise greater self-compassion [91]. There was also some evidence of upward social comparison (hope for recovery) [28] and downward social comparison (gaining perspective and a sense of progress) [37, 92].

Many mothers found it beneficial to hear peer experiences and advice about self-care and coping with mental health and parenting issues. This could be understood through the lenses of social learning theory [93] and informational social support leading to problem-focused coping [44]. The particular credibility of peers was sometimes highlighted, their experiential knowledge [42] contrasted with health professionals whose suggestions might be unrealistic.

By highlighting contexts as an integral part of the causal pathway, the realist approach helps to explain why peer support appeals to some mothers but not others, and ‘works’ for some mothers but not others. There was, however, no evidence that the mechanisms worked differently for first time mothers compared to mothers who already had a child, for mothers with a history of mental health difficulties compared to mothers with no previous history, or for mothers with a partner compared to single mothers. There was also no evidence that there was any ‘dose’ of peer support that was more or less effective than any other, nor that any particular mental health scale cut-off for the lower or upper boundary of access to the peer support affected the mechanisms or outcomes. It was suggested by Shorey and Ng [79] that peer support might be particularly useful for mothers in conservative Asian cultures, but the evidence in this review was that mothers in other cultural contexts (in the UK, USA and Canada) also found it difficult to talk to their social network about their mental health difficulties, and the mechanisms related to non-judgmental, empathetic listening appeared to be cross-cultural.

There was some evidence that shared experience of perinatal mental health difficulties alone might not always be a sufficient basis for some peer support mechanisms, particularly empathy and the formation of social relationships. One-to-one peer support relationships could be strengthened by careful matching based on similarities in background and interests. There was not enough detail to draw clear conclusions about the extent to which mothers valued social or cultural similarity within a peer support group. Although the majority of participants in most studies tended to be socio-economically advantaged and educated, there were also examples of interventions that had successfully devised ways to overcome practical barriers to access (theory 10).

The different formats of peer support had the potential to engage and benefit mothers in different ways. One-to-one telephone support offered the possibility of a convenient, flexible, anonymous peer support relationship that did not incur travel costs or time, and thus might improve access for disadvantaged mothers; the attraction of anonymity is consistent with research into telephone peer support in other sensitive contexts [94]. There were, however, limitations on the strength of relationships that could be formed by telephone, and support by telephone may have had a weaker effect on loneliness. In the UK, where there was (at that time) no culture of telehealth, there was comparatively low take-up of telephone peer support when offered, and high drop-out [82]. One-to-one face-to-face support had the potential for stronger relationships and more in-depth disclosure, but would not suit mothers who valued anonymity, and might have travel implications for the mother if meetings were not at her home. One-to-one support in general might have social costs for mothers who did not have the confidence to break appointments that they did not want to keep, and could have some risks if the peer supporter was insufficiently trained in how to have supportive conversations.

Group support offered a wider range of peer experiences with greater potential for social comparison, sharing coping strategies and the opportunity for reciprocal support, although there was unexpectedly little evidence reported of mothers benefiting through the opportunity to help others at peer support groups [45]. Group support might create the opportunity for new friendships, particularly benefiting mothers who were socially isolated. It offered some mothers structure, a reason to leave their home, and a break from their children, although it also involved travel costs and time. Group support was not suitable for mothers who lacked the social confidence to attempt a group situation, and if it was not well facilitated had risks of several negative mechanisms when mothers compared themselves to others, felt sad about others’ situations, and did not feel heard or validated, as also identified in groups more generally by Morrell et al. [13]. None of the included interventions offered mothers a choice of one-to-one or group support, so there was no evidence about how these might compare in practice for individuals.

There was a clear overlap between the theories identified for group peer support and the social psychological mechanisms active in group psychotherapy [18, 95], and between the theories identified for one-to-one support and the core conditions of humanistic therapy – genuineness, full acceptance and empathy [90]. There was also a substantial overlap between the mechanisms related to the use of volunteers to deliver one-to-one mental health peer support in these interventions, and those found in other forms of one-to-one volunteering in the perinatal period [96, 97].

There was an unresolved tension between the desire in some interventions to train peer supporters as little as possible to avoid ‘professionalising’ their role or overtaxing the volunteers [58, 66], and the benefits of a more comprehensive training in active listening and support skills [96], although it was notable that there was a trend for training to be lengthened when models were replicated. This relative lack of training may have contributed to some negative theories when volunteers gave directive advice in the belief that their role was to ‘fix’ or ‘solve’ the mother’s problems for her, failed to validate mothers’ problems, and overshared their own experiences. Most of the evidence for negative C-M-Os, however, came from interactions between mothers during group support, rather than between trained volunteers and mothers. Given the additional benefits of group support for some mothers, this emphasises the importance of ensuring that group leaders have the facilitation skills to anticipate and mitigate negative mechanisms.

It has been argued that the effectiveness of peer support in mental health services should be evaluated with consistent measures that meaningfully capture what it is actually likely to achieve, such as improvements in subjective distress and psychosocial outcomes including hope and optimism, life satisfaction, wellness, confidence, connectedness, community empowerment and social support [98–101]. Aside from appropriate outcome measures, effectiveness research for community-based peer support is additionally complicated by the challenge of identifying appropriate methodologies to capture impact in the context of fluid attendance at small scale groups, support based on spontaneous human relationships, and ethical opposition to randomising people to receive no support [24, 87, 102]. This review found limited randomised controlled trial evidence for a short-term, statistically significant reduction in symptoms of postnatal depression after a 4–12 week period of one-to-one [62, 103] or group peer support [78], but not for anxiety, and there was no evidence about antenatal depression or other mental health difficulties.

There was no evidence to connect these improvements in depressive symptomology to any particular C-M-O configuration. The detachment of these ‘hard’ mental health outcomes from the proximate outcomes reported through qualitative evidence and questionnaires mirrors the complexity of the concept of ‘recovery’ in mental health [104, 105]. It is plausible that, through the activation of one or more of the C-M-Os, for some mothers peer support has a direct impact on recovery from the symptoms of perinatal mental health difficulties as measured by mental health scores. However, it might be also possible for a mother to have improved her subjective wellbeing - through reduced feelings of guilt, shame and alienation, and increased ability to cope with parenting and her mental health difficulties – while remaining depressed or anxious as measured by a screening questionnaire or clinical interview; this could be considered recovery ‘within’ mental health difficulties.

This latter view is consistent with Rosenberg’s analysis of support groups as offering “comfort rather than cure” (p.178) [30]. It was, however, clear from the use of mental health scales by many of the interventions in this review that community-based peer support programmes may seek to demonstrate that they can also offer a form of ‘cure’. This may be influenced by the requirements of funders as well as a desire to show mothers and potential referrers that the peer support is effective. These two different versions of ‘outcomes’ reflect the paradox inherent in the ‘normalisation’ of perinatal mental health difficulties through peer support, as noted by Taylor [23]: mothers seek out and benefit from lateral social comparison which ‘normalises’ their current difficult emotions, but at the same time seek out and benefit from upward social comparison in the hopeful stories of mothers who have returned to a more mainstream ‘normality’ where they no longer have those difficult emotions. Connecting contexts, mechanisms and outcomes provides a theoretical basis for understanding the differences in how mothers respond to this and other aspects of peer support.

It remains unresolved as to how an outcomes evaluation of flexible and needs-led community-based perinatal mental health peer support can be simultaneously scientifically rigorous and consistent with peer support principles, and how to generate evidence about the relative contribution of different C-M-Os to improvements in mental health symptomology. To enable some consistency and comparability across third sector perinatal mental health peer support programmes, it would beneficial for peer support providers and researchers to discuss collectively the questions of which mental health, personal recovery and other outcomes should be measured, how they should be measured, whether there is a minimum amount of peer support that should be experienced before impact is judged, and how this can be achieved in a flexible programme.

Future research could investigate how mothers who do not want to attend a mental health programme can be enabled to benefit from peer support *mechanisms* in other settings, and identify C-M-Os for fathers and other parents with perinatal mental health difficulties. Future research could also explore how the organisation of third sector perinatal mental health peer support may affect C-M-Os and effectiveness, for example organisational ethos and leadership, differences between peer support as a freestanding offer or offered alongside psychological therapy from the same organisation, and the C-M-Os where a third sector organisation is commissioned to provide peer support inside a professional mental health service.

### Strengths and limitations

It was a strength of this review that it was able to combine studies from a wide variety of qualitative and quantitative methodologies and descriptions of process. The methodological quality of included documents was extremely variable, but this review was strengthened by the inclusive realist approach of examining every source critically for the trustworthy pieces of information it could yield. It was also a strength that the review was informed by discussions and debate with women with lived experience of perinatal mental health difficulties, providers and commissioners of community-based peer support, at multiple events during the different stages of the review. It was a limitation that responses were received from only three of the 11 third sector programmes contacted. None of the included studies were realist. Because non-realist authors do not necessarily investigate or report aspects of their programme that a realist would see as contextual factors or mechanisms, lack of evidence for programme theories could be an artefact of limited reporting or insight [106].

## Conclusions

Peer support should not be seen as a low-cost substitute for professional support when it is needed, but as a worthwhile complementary and skilled intervention that can benefit mothers in complex ways, including recovery from perinatal mental health symptoms for some, and subjectively meaningful improvements in wellbeing for others. Mothers with perinatal mental health difficulties are heterogeneous in their backgrounds, personalities, social situations, resources, and needs, and these personal contextual factors as well as wider social contextual factors affect mothers’ beliefs about the benefits of peer support, and thus their decision to use it as well as their ability to use it. Once a mother takes up peer support, there are multiple mechanisms which may be activated to produce positive or negative outcomes in different contexts; the evidence for positive mechanisms and outcomes being much stronger than the negative. This review can be used by providers, commissioners and evaluators to understand the value and complexity of the contribution of community-based peer support programmes to improving the emotional wellbeing of mothers with perinatal mental health difficulties. It can assist in identifying the range of benefits for different mothers, avoiding the potential risks, and devising ways to reach mothers who do not currently engage with peer support.

### Electronic supplementary material

Below is the link to the electronic supplementary material.

Supplementary Material 1

Supplementary Material 2

Supplementary Material 3

Supplementary Material 4

Supplementary Material 5

Supplementary Material 6

Supplementary Material 7

## Data Availability

All data generated or analysed during this study are included in this published article [and its supplementary information files].
