# Supplementary material for: Community-based perinatal mental health peer support: a realist review
Source: BMC Pregnancy Childbirth. 2023 Aug 9;23:570. doi: 10.1186/s12884-023-05843-8 (PMC10410814; doi:10.1186/s12884-023-05843-8)
Supplement: Supplementary file 1 — Supplementary Material 1 [file 12884_2023_5843_MOESM1_ESM.docx]

**Construction of the initial theoretical model: evidence sources for potential contextual factors affecting take-up and impact of community-based perinatal mental health peer support**

| **Theory #** | **Potential contextual factor affecting take-up and/or impact of peer support** | | **Sources – references are separate from main article** |
| --- | --- | --- | --- |
|  | **Society/ community level** | **Individual level** |  |
|  | **The ‘myth’ of motherhood and stigma of mental illness** | | |
| 1-3, 14-17 | Cultural narratives of idealised motherhood |  | [1-5] |
| 1-3, 14-17 | Stigma of mental illness |  |  |
| 1, 14-17 |  | Mother labels herself negatively as a uniquely abnormal 'bad' mother | [1, 5-25] |
|  | **Withdrawal from family and friends** | | |
| 2, 14-17 |  | Mother hides feelings from partner, family & friends and cannot meet needs for authenticity in relationships | [5, 6, 12, 14, 16, 19, 26-28] |
| 2, 3, 14-17, 22 |  | Mother lacks a social network to migration or homelessness | [28] |
|  | **Withdrawal from other mothers** | | |
| 3, 14-17 | Expectation that new mothers will meet social support needs through other new parents |  | [12, 22] |
| 3 |  | Mother avoids new parent groups as these make her feel worse | [12, 15, 16, 22, 23] [12, 21] |
|  | **Avoiding professional intervention and referral pathways** | | |
| 4,5 | Primary health professionals have limited training on perinatal mental health difficulties and limited time |  | [29, 30] |
| 4 |  | Mother conceals symptoms from professionals – fear of judgment, consequences, lack of understanding / empathy | [1, 14, 19, 24, 26, 27, 31-34] |
| 5 |  | Mother trusts health professionals | [35] |
|  | **Diverse conceptions of perinatal mental health difficulties and the merits of talking about them** | | |
| 6 | Different conceptions of mental health difficulties and appropriate response | Mother has a personal conception of cause and meaning of perinatal mental health difficulties | [1, 5, 7, 11, 14, 17, 19, 24, 26, 27, 31-33, 36-49] |
| 7 | Public health campaigns promote message ‘it's good to talk’, but there are differences in the acceptability of talking to outsiders | Mother believes it is useful and acceptable to talk about mental health difficulties | [5, 24, 47, 48, 50-52] |
|  | **Cultural homogeneity or heterogeneity** | | |
| 8 |  | Mother has a preference for cultural homogeneity or heterogeneity | [24, 48, 53-56] |
|  | **Peer support as a poor relation to professional support** | | |
| 9, 19 | Limited access to perinatal mental health support, including long waiting lists | Mother actually wants psychological therapy | [34, 57] |
|  | **Lack of time and money** | | |
| 10, 23 | Social norm that mother is primarily responsible for meeting baby's needs alongside domestic responsibilities and other work | Mother has resources of time and/or money to invest in meeting her own needs | [5, 49, 58] |
|  | **Nature and severity of the mental health difficulties** | | |
| 11, N7-N8 |  | Mother has low social confidence and preference for group or one to on support | [28, 35] |
| 20 |  | Mother is sufficiently well to give as well as benefit from receiving peer support | [59, 60] |
| 21, N1-N4, |  | Mother has low self-esteem and low internal locus of control | [61] |
|  | **Peer support volunteers** | | |
| 13 | Pool of suitable potential volunteers exists in local community |  | [35] |

**Key**

Theory numbers refer to the final theoretical model – see additional files 5-7

**REFERENCES FOR ADDITIONAL FILE 1**

1. Knudson-Martin C, Silverstein R: **Suffering in silence: a qualitative meta-data-analysis of postpartum depression**. *J Marital Fam Ther* 2009, **35**(2):145-158.

2. Stoppard J: **Understanding Depression: Feminist Social Constructionist Approaches**. London: Routledge; 2000.

3. Hays S: **The cultural contradictions of motherhood**. New Haven: Yale University Press; 1996.

4. Hall P: **Mothering mythology in the late twentieth century: science, gender lore and celebratory narrative**. *Canadian Woman Studies* 1998, **18**(2/3):59.

5. Schmied V, Black E, Naidoo N, Dahlen HG, Liamputtong P, Foster AM: **Migrant women’s experiences, meanings and ways of dealing with postnatal depression: A meta-ethnographic study**. *PLoS One* 2017, **12**(3):e0172385-e0172385.

6. Bilszta J, Ericksen J, Buist A, Milgrom J: **Women's experience of postnatal depression - beliefs and attitudes as barriers to care**. *Aust J Adv Nurs* 2010, **27**(3):44-54.

7. Ali E: **Women's experiences with postpartum anxiety disorders: a narrative literature review**. *International journal of women's health* 2018, **10**:237-249.

8. Coates R, Ayers S, de Visser R: **Women's experiences of postnatal distress: a qualitative study**. *BMC Pregnancy Childbirth* 2014, **14**:359.

9. Evans K, Morrell CJ, Spiby H: **Women’s views on anxiety in pregnancy and the use of anxiety instruments: a qualitative study**. *J Reprod Infant Psychol* 2017, **35**(1):77-90.

10. Haga SM, Lynne A, Slinning K, Kraft P: **A qualitative study of depressive symptoms and well-being among first-time mothers**. *Scand J Caring Sci* 2012, **26**(3):458-466.

11. Highet N, Stevenson AL, Purtell C, Coo S: **Qualitative insights into women's personal experiences of perinatal depression and anxiety**. *Women & Birth* 2014, **27**(3):179-184.

12. Mauthner NS: **Postnatal depression: The significance of social contacts between mothers**. *Womens Stud Int Forum* 1995, **18**(3):311-323.

13. Beck CT: **Postpartum depression: a metasynthesis**. *Qual Health Res* 2002, **12**(4):453-472.

14. Staneva AA, Wigginton B: **The happiness imperative: Exploring how women narrate depression and anxiety during pregnancy**. *Fem Psychol* 2018, **28**(2):173-193.

15. Beck CT: **Teetering on the edge: a substantive theory of postpartum depression**. *Nurs Res* 1993, **42**(1):42-48.

16. Bennett HA, Boon HS, Romans SE, Grootendorst P: **Becoming the best mom that I can: women's experiences of managing depression during pregnancy -a qualitative study**. *BMC Womens Health* 2007, **7**(1):13.

17. Ugarriza DN: **Postpartum Depressed Women's Explanation of Depression**. *J Nurs Scholarsh* 2002, **34**(3):227-233.

18. Choi P, Henshaw C, Baker S, Tree J: **Supermum, superwife, supereverything: performing femininity in the transition to motherhood**. *J Reprod Infant Psychol* 2005, **23**(2):167-180.

19. Patel S, Wittkowski A, Fox JRE, Wieck A: **An exploration of illness beliefs in mothers with postnatal depression**. *Midwifery* 2013, **29**(6):682-689.

20. Raymond JE: **'Creating a safety net': Women's experiences of antenatal depression and their identification of helpful community support and services during pregnancy**. *Midwifery* 2009, **25**(1):39-49.

21. Jones CCG, Jomeen J, Hayter M: **The impact of peer support in the context of perinatal mental illness: A meta-ethnography**. *Midwifery* 2014, **30**(5):491-498.

22. Scrandis DA: **Normalizing Postpartum Depressive Symptoms With Social Support**. *J Am Psychiatr Nurses Assoc* 2005, **11**(4):223-230.

23. Tammentie T, Paavilainen E, Åstedt-Kurki P, Tarkka M: **Family dynamics of postnatally depressed mothers - discrepancy between expectations and reality**. *J Clin Nurs* 2004, **13**(1):65-74.

24. Edge D, Rogers A: **Dealing with it: Black Caribbean women's response to adversity and psychological distress associated with pregnancy, childbirth, and early motherhood**. *Soc Sci Med* 2005, **61**(1):15-25.

25. Abrams LS, Curran L: **Maternal identity negotiations among low-income women with symptoms of postpartum depression**. *Qual Health Res* 2011, **21**(3):373-385.

26. Franks WLM, Crozier KE, Penhale BLM: **Women’s mental health during pregnancy: A participatory qualitative study**. *Women & Birth* 2017, **30**(4):e179-e187.

27. McIntosh J: **Postpartum depression: women's help-seeking behaviour and perceptions of cause**. *J Adv Nurs* 1993, **18**(2):178-184.

28. McLeish J, Redshaw M: **Mothers' accounts of the impact on emotional wellbeing of organised peer support in pregnancy and early parenthood: a qualitative study**. *BMC Pregnancy Childbirth* 2017, **17**:1-14.

29. Khan L: **Falling through the gaps: perinatal mental health and general practice**. In*.* London: Centre for Mental Health; 2015.

30. Bayrampour H, Hapsari AP, Pavlovic J: **Barriers to addressing perinatal mental health issues in midwifery settings**. *Midwifery* 2018, **59**:47-58.

31. Iles J, Pote H: **Postnatal posttraumatic stress: A grounded theory model of first-time mothers’ experiences**. *J Reprod Infant Psychol* 2015, **33**(3):238-255.

32. Edge D, MacKian SC: **Ethnicity and mental health encounters in primary care: help-seeking and help-giving for perinatal depression among Black Caribbean women in the UK**. *Ethn Health* 2010, **15**(1):93-111.

33. Morrow M, Smith JE, Lai Y, Jaswal S: **Shifting Landscapes: Immigrant Women and Postpartum Depression**. *Health Care Women Int* 2008, **29**(6):593-617.

34. Royal College of Obstericians and Gynaecologists: **Maternal Mental Health - Women's Voices**. In*.* Retrieved 30.10.21, from <https://www.rcog.org.uk/for-the-public/rcog-engagement-listening-to-patients/maternal-mental-health-womens-voices/>; 2017.

35. McLeish J, Baker L, Connolly H, Davis H, Pace C, Suppiah C: **Volunteering and early childhood outcomes: A review of the evidence**. In*.* London: Parents 1st; Institute for Voluntary Action Research; 2016.

36. Mauthner N: **"Feeling low and feeling really bad about feeling low": Women's experiences of motherhood and postpartum depression**. *Can Psychol* 1999, **40**(2):143-161.

37. Nicolson P: **Explanations of post natal depression: Structuring knowledge of female psychology**. *Research on Language and Social Interaction* 1991, **25**(1-4):75-96.

38. Kleinman A, Eisenberg L, Good B: **Culture, illness, and care: clinical lessons from anthropologic and cross-cultural research**. *Ann Intern Med* 1978, **88**(2):251-258.

39. Baines T, Wittkowski A: **A systematic review of the literature exploring illness perceptions in mental health utilising the self-regulation model**. *J Clin Psychol Med Settings* 2013, **20**(3):263-274.

40. Baines T, Wittkowski A, Wieck A: **Illness perceptions in mothers with postpartum depression**. *Midwifery* 2013, **29**(7):779-786.

41. Small R, Brown S, Lumley J, Astbury J: **Missing voices: What women say and do about depression after childbirth**. *J Reprod Infant Psychol* 1994, **12**(2):89-103.

42. Wittkowski A, Zumla A, Glendenning S, Fox JRE: **The experience of postnatal depression in South Asian mothers living in Great Britain: a qualitative study**. *J Reprod Infant Psychol* 2011, **29**(5):480-492.

43. Watson H, Harrop D, Walton E, Young A, Soltani H: **A systematic review of ethnic minority women's experiences of perinatal mental health conditions and services in Europe.(Research Article)**. *PLoS One* 2019, **14**(1):e0210587.

44. Coates R, de Visser R, Ayers S: **Not identifying with postnatal depression: a qualitative study of women's postnatal symptoms of distress and need for support**. *J Psychosom Obstet Gynaecol* 2015, **36**(3):114-121.

45. Abrams LS, Curran L: **“And You're Telling Me Not to Stress?” a Grounded Theory Study of Postpartum Depression Symptoms among Low-Income Mothers**. *Psychol Women Q* 2009, **33**(3):351-362.

46. Gardner PL, Bunton P, Edge D, Wittkowski A: **The experience of postnatal depression in West African mothers living in the United Kingdom: a qualitative study**. *Midwifery* 2014, **30**(6):756-763.

47. Oates MR, Cox JL, Neema S, Asten P, Glangeaud-Freudenthal N, Figueiredo B, Gorman LL, Hacking S, Hirst E, Kammerer MH *et al*: **Postnatal depression across countries and cultures: a qualitative study**. *Br J Psychiatry Suppl* 2004, **46**:s10-16.

48. Parvin A, Jones CE, Hull SA: **Experiences and understandings of social and emotional distress in the postnatal period among Bangladeshi women living in Tower Hamlets**. *Fam Pract* 2004, **21**(3):254-260.

49. Templeton L, Velleman R, Persaud A, Milner P: **The experiences of postnatal depression in women from black and minority ethnic communities in Wiltshire, UK**. *Ethn Health* 2003, **8**(3):207-221.

50. **Britain Get Talking** [<https://youngminds.org.uk/get-involved/campaign-with-us/britain-get-talking/>]

51. **How to approach difficult conversations** [<https://www.headstogether.org.uk/talking-mental-health-guide/>]

52. Burr J, Chapman T: **Contextualising experiences of depression in women from South Asian communities: a discursive approach**. *Sociol Health Illn* 2004, **26**(4):433-452.

53. Masood Y, Lovell K, Lunat F, Atif N, Waheed W, Rahman A, Mossabir R, Chaudhry N, Husain N: **Group psychological intervention for postnatal depression: a nested qualitative study with British South Asian women**. *BMC Womens Health* 2015, **15**:109-109.

54. McLeish J, Redshaw M: **Peer support during pregnancy and early parenthood: a qualitative study of models and perceptions**. *BMC Pregnancy Childbirth* 2015, **15**(1):257.

55. Billsborough J, Currie R, Gibson S, Gillard S, Golightley S, Kotecha-Hazzard K, Mesaric A, Mohammed S, Pinfold V, Sweet D *et al*: **Evaluating the Side by Side peer support programme**. In*.* London: St George’s, University of London and McPin Foundation; 2017.

56. Faulkner A, Sadd J, Hughes A, Thompson S, Nettle M, Wallcraft J, Collar J, de la Haye S, McKinley S: **Mental health peer support in England: Piecing together the jigsaw**. In*.* London: MIND; 2013.

57. **All about us** [<https://cocoonfamilysupport.org/about-cocoon>]

58. Goodman JH: **Women's attitudes, preferences, and perceived barriers to treatment for perinatal depression**. *Birth* 2009, **36**(1):60-69.

59. Frank A: **Just Listening: Narrative and Deep Illness**. *Families, Systems & Health* 1998, **16**(3):197-212.

60. Rennick-Egglestone S, Ramsay A, McGranahan R, Llewellyn-Beardsley J, Hui A, Pollock K, Repper J, Yeo C, Ng F, Roe J *et al*: **The impact of mental health recovery narratives on recipients experiencing mental health problems: Qualitative analysis and change model**. *PLoS One* 2019, **14**(12):e0226201.

61. Bogart LM, Helgeson VS: **Social Comparisons Among Women With Breast Cancer: A Longitudinal Investigation1**. *J Appl Soc Psychol* 2000, **30**(3):547-575.
