# Supplementary material for: Community-based perinatal mental health peer support: a realist review
Source: BMC Pregnancy Childbirth. 2023 Aug 9;23:570. doi: 10.1186/s12884-023-05843-8 (PMC10410814; doi:10.1186/s12884-023-05843-8)
Supplement: Supplementary file 2 — Supplementary Material 2 [file 12884_2023_5843_MOESM2_ESM.docx]

**Construction of the initial theoretical model: mid-range theories used as evidence sources for potential positive and negative mechanisms and outcomes for mothers using peer support**

| Theory # | **Potential positive mechanisms** | | **Potential positive outcomes** | **Evidence source - psychological theories [main paper references]** |
| --- | --- | --- | --- | --- |
|  | **What happens during peer support** | **Reasoning or reaction of mother** |  |  |
| 14 | **Mother talks honestly and is listened to empathetically** | Feeling understood | Emotional release: able to share true self | Emotional social support [40]  Humanistic therapy principles [87] |
| 15 | **Mother talks honestly and is listened to non-judgementally** | Feeling accepted by others, leading to self-acceptance | Improved self-esteem / self-concept  Mother gains confidence to disclose to partner/family/friends and authentic relationships are restored  Mother gains confidence to attend 'normal' new parent groups | Social integration or network social support [40]  Humanistic therapy principles [87]  Overcoming stigma [38]  Emotional social support [40] Self-compassion [88]  Attachment theory [43] |
| 16 | **Peers give positive feedback about her feelings and actions** | Feeling affirmed | Improved self-esteem / self-concept | Esteem social support [40]  Perception-focused coping assistance [41]  Overcoming stigma [38] |
| 17 | **Peers talk about their own perinatal mental health and parenting challenges** | Mental health and parenting challenges are normalised  Compares herself to peers who are now well  Compares herself to peers who are less well  Compassion for others promotes self-compassion | Improved self-esteem / self-concept  Hope for recovery  Sense of perspective  Improved self-concept | Normative narrative community [30] Lateral social comparison [37] Overcoming stigma [38]  Esteem social support [40]  Perception-focused coping assistance[41] Experiential knowledge [39]  Upward social comparison [37]  Downward social comparison [37]  Self-compassion [88] |
| Theory # | **Potential positive mechanisms** | | **Potential positive outcomes** | **Evidence source - psychological theories** |
|  | **What happens during peer support** | **Reasoning or reaction of mother** |  |  |
| 18 | **Peers share ideas about self-care, coping with perinatal mental health, parenting, medication, mental health services & community services** | Mother gains information she finds credible and encouragement to try new things | Increased coping strategies and lower stress  Increased take-up of mental health services  Increased use of other community services | Experiential knowledge [39] Informational social support [40]  Problem-focused coping assistance [41] |
| 19 | **Peers use therapeutic techniques such as reframing, challenging negative cognitions** | Mother experiences therapy-lite  Mother gains access to techniques she can try | Increased coping with perinatal mental health difficulties |  |
| 20 | **Opportunity for reciprocal support** | Mother offers support to other mothers in a group | Self-esteem Finding meaning in own experiences | Helper therapy [42] |
| 21 | **Programme offers support from volunteers who are not paid for their time** | Mother experiences herself as worth another person’s time | Self-esteem  Feels cared about |  |
| 22 | **Same peers attend group over time, or 1:1 peer supporter is well matched.**  **Peer support is for mothers whose mental health difficulties are specifically connected to having a baby, and are mild-to-moderate.** | Mother forms meaningful relationships (that may continue outside group) | Reduced loneliness Increased social network | Social integration or network social support [40]  Attachment theory [43] |
| Theory # | **Potential negative mechanisms** | | **Potential negative outcomes** | **Evidence source - psychological theories** |
|  | **What happens during peer support** | **Reasoning or reaction of mother** |  |  |
| N1 | **Peers talk about their own perinatal mental health and parenting challenges but do not validate others’ experiences and feelings**  **Programme has inclusive approach and the mothers are not similar in background or mental health experience** | Mother feels unvalidated, abnormal | Increased sense of abnormality and shame  Loss of authenticity | Lateral social comparison [37]  Normative narrative community [30] |
| N2 | **Peers talk about their own perinatal mental health and parenting challenges** | Mother feels bad about not coping when others are worse, or fears getting worse | Self-criticism / reduced emotional wellbeing | Downward social comparison [37] |
| N3 | **Peers talk about their own perinatal mental health and parenting challenges** | Mother feels discouraged that others are getting well more quickly | Self-criticism / reduced emotional wellbeing | Upward social comparison [37] |
| N4 | **Peers focus on sharing negative feelings and experiences; no use of therapeutic techniques.**  **Group does not have effective facilitation or peer supporters are not well selected/ trained** | Mother responds to others’ negativity or distress with own negativity and distress | Reduced emotional wellbeing, anxiety over own recovery and sadness over others’ suffering | Lateral social comparison [37]  Helper therapy [42] |
| Theory # | **Potential negative mechanisms** | | **Potential negative outcomes** | **Evidence source - psychological theories** |
|  | **What happens during peer support** | **Reasoning or reaction of mother** |  |  |
| N8 | **Peer support becomes a safe bubble but mother is unable to move beyond it to authentic relationships with non-peers** | Mother is distressed about ending of support that she relies on. | Anxiety about ending.  Loss of social support.  Grief and sense of loss after ending. |  |
| N9 | **Attempts at friendship unsuccessful** | Reinforces sense of social failure | Reduced social confidence and self-esteem | Social integration or network social support [40] |
| N  10 | **Peers share unhelpful ideas about self-care, coping with perinatal mental health, parenting, medication, mental health services**  **Group does not have effective facilitation** | Mother is influenced by poor advice from group members, not redressed by facilitator | Lose confidence in mental health services, use ineffective or harmful coping strategies | Experiential knowledge [39] |

**Key**

Theory numbers refer to the final theoretical model – see additional files 6-7
