# Supplementary material for: Community-based perinatal mental health peer support: a realist review
Source: BMC Pregnancy Childbirth. 2023 Aug 9;23:570. doi: 10.1186/s12884-023-05843-8 (PMC10410814; doi:10.1186/s12884-023-05843-8)
Supplement: Supplementary file 3 — Supplementary Material 3 [file 12884_2023_5843_MOESM3_ESM.docx]

# Quality assessment of studies included in realist review

Quality assessment criteria from the Mixed Methods Appraisal Tool, version 2018 [52], were applied to eight qualitative studies (Table 1.S), seven reporting the results of randomised controlled trials (Table 2.S), five reporting the results of non-randomised quantitative studies (Table 3.S), three quantitative descriptive studies (Table 4.S), and two mixed methods studies (Table 5.S, Table 6.S). They were not applied to an additional four papers describing process only [57, 67, 73, 74]. For the purpose of this assessment, studies reporting answers to open text questions on questionnaires, where these data were not analysed but only used illustratively, were classed as quantitative studies (and appear in the table relevant to their quantitative methodology).

Note: Answer options are Yes, No, Can’t tell (CT).

Table 1.S Quality assessment of qualitative studies

| **Authors** | **Clear research questions or objectives** | **Collected data address the research question** | **1.1 Is the qualitative approach appropriate to answer the research question?** | **1.2 Are the qualitative data collection methods adequate to address the research question?** | **1.3 Are the findings adequately derived from the data?** | **1.4 Is the interpretation of results sufficiently substantiated by data?** | | **1.5 Is there coherence between qualitative data sources, collection, analysis and interpretation?** | **Comments** |
| --- | --- | --- | --- | --- | --- | --- | --- | --- | --- |
| **Anderson (2013)** | Yes | Yes | Yes | Yes | Yes | No | Yes | | No details about groups or population.  Limited use of quotation. |
| **Carter et al (2018)** | Yes | Yes | Yes | Yes | Yes | Yes | Yes | | Thematic analysis weak. |
| **Carter et al (2019)** | Yes | Yes | Yes | Yes | Yes | No | Yes | | Member checking done.  Thematic analysis weak.  Limited use of quotation. |
| **Duskin (2005)** | Yes | Yes | Yes | Yes | Yes | Yes | Yes | | In depth, theorised analysis with strong use of quotation.  No justification given for low number of interviewees.  Researcher was also group leader, but analysis audited by independent person. |
| **Letourneau et al (2015)** | Yes | Yes | Yes | Yes | Yes | Yes | Yes | | Useful for context. |
| **Ludwick (2017)** | Yes | No | No | No | No | No | No | | No data collection apart from researcher’s notes.  No analysis.  Useful for failure of group telephone support. |
| **Montgomery et al (2012)** | Yes | Yes | Yes | Yes | Yes | Yes | Yes | | Analysis focused on stories that fit recovery narrative.  Quotes not attributed. |
| **Shorey and Ng (2019)** | Yes | Yes | Yes | Yes | Yes | Yes | Yes | | Thematic analysis limited.  Useful for context. |
| **Lynch (2019)** | Yes | Yes | Yes | Yes | CT | No | Yes | | Purpose is report on social return on investment. |

Table 2.S Quality assessment of quantitative studies reporting results of randomised controlled trials

| **Authors** | **Clear research questions or objectives** | **Collected data address the research question** | **2.1. Is randomisation appropriately performed?** | **2.2. Are the groups comparable at baseline?** | **2.3. Are there complete outcome data? (defined as 80%)** | | **2.4. Are outcome assessors blinded to the intervention provided?** | **2.5 Did the participants adhere to the assigned intervention?** | **Comments** |
| --- | --- | --- | --- | --- | --- | --- | --- | --- | --- |
| **Chen et al (2000)** | Yes | Yes | Yes | Yes | Yes | CT | | Yes | 4/34 (12%) dropped out.  Outcome data for 60/64 (94%).  Discussion introduces women’s views, but source of these is unclear. |
| **Dennis (2003)** | Yes | Yes | Yes | CT | Yes | Yes | | CT | No drop-out.  Outcome data for 43/44 (98%).  Analysis controlled for baseline differences.  Used intention to treat analysis.  Insufficiently powered for secondary outcomes. |
| **Dennis et al (2009)** | Yes | Yes | Yes | Yes (from 2010 paper) | Yes | Yes | | CT | Intervention initiated for 328/349 (94%).  Outcome data for 600/701 (85%) at 24 weeks.  Analysis controlled for baseline differences. |
| **Field et al (2013a)** | Yes | Yes | CT | No | Yes | CT | | Yes | 4/48 (8%) dropped out across both conditions.  Outcome data for 44/48 (92%) |
| **Field et al (2013b)** | Yes | Yes | CT | Yes | Yes | Yes | | Yes | 11/92 (12%) dropped out.  Outcome data for 78/92 (85%) |
| **Gjerdingen et al (2013)** | Yes | Yes | Yes | No | Yes | No | | CT | 2/39 (5%) dropped out.  Outcome data for 36/39 (92%).  Analysis controlled for baseline differences. |
| **Shorey et al (2019)** | Yes | Yes | Yes | CT | Yes | Yes | | CT | No drop out.  Outcome data for 113/ 138 (82%).  Used intention to treat analysis.  Analysis controlled for baseline differences. |

Table 3.S Quality assessment of quantitative studies reporting results of non-randomised studies

| **Authors** | **Clear research questions or objectives** | **Collected data address the research question** | **3.1. Are the participants representative of the target population?** | **3.2. Are measurements appropriate regarding both the outcome and intervention (or exposure)?** | **3.3. Are there complete outcome data?** | **3.4. Are the confounders accounted for in the design and analysis?** | **3.5. During the study period, is the intervention administered (or exposure occurred) as intended?** | **Comments** |
| --- | --- | --- | --- | --- | --- | --- | --- | --- |
| **Acacia Family Support (2019)** | Yes | Yes | CT | Yes | No | No | CT | Outcome data for 159/535 (29%)  Limited reporting of statistical results. |
| **Eastwood et al (1995)** | Yes | Yes | CT | Yes | No | No | No | 5/13 (38%) dropped out.  Outcome data on 8/13 (62%)  Limited reporting of statistical results.  Researcher’s observations included. |
| **Letourneau et al (2016)** | Yes | Yes | CT | Yes | No | No | CT | 30/64 (47%) dropped out / lost to follow up.  Outcome data for 34/64 (53%). |
| **Prevatt et al (2018)** | Yes | Yes | CT | Yes | No | Yes | CT | Outcome data for 25/45 (56%). |
| **Fairbairn and Kitchener (2020)** | Yes | Yes | CT | Yes | No | No | CT | Outcome data for 53/126 (42%).  Very limited reporting of statistical results. |

Table 4.S Quality assessment of quantitative descriptive studies

| **Authors** | **Clear research questions or objectives** | **Collected data address the research question** | **4.1. Is the sampling strategy relevant to address the research question?** | **4.2. Is the sample representative of the target population?** | **4.3. Are the measurements appropriate?** | **4.4. Is the risk of nonresponse bias low?** | **4.5. Is the statistical analysis appropriate to answer the research question?** | **Comments** |
| --- | --- | --- | --- | --- | --- | --- | --- | --- |
| **Dennis (2010)** | Yes | Yes | Yes | Yes | Yes | CT | Yes | 221/349 (63%) intervention mothers responded. |
| **Dennis (2013)** | Yes | Yes | Yes | Yes | Yes | CT | Yes | 121/175 (69%) volunteers who had supported a mother responded. |
| **Pitts (1999)** | Yes | Yes | Yes | Yes | Yes | CT | No analysis done | 34/48 (71%) mothers responded.  Large sample of open text answers quoted as list. |

Table 5.S Mixed methods studies combining RCT and qualitative methods (part 1)

| **Authors** | **Clear research questions or objectives** | **Collected data address the research question** | **1.1 Is the qualitative approach appropriate to answer the research question?** | **1.2 Are the qualitative data collection methods adequate to address the research question?** | **1.3 Are the findings adequately derived from the data?** | **1.4 Is the interpret-ation of results sufficiently substant-iated by data?** | **1.5 Is there coherence between qualitative data sources, collection, analysis and interpretat-ion?** | **2.1. Is randomis-ation appropriately performed?** | **2.2. Are the groups compar-able at baseline?** | **2.3. Are there complete outcome data? (defined as 80%)** | **2.4. Are outcome assessors blinded to the intervent-ion provided?** | **2.5 Did the particip-ants adhere to the assigned interven-tion?** |
| --- | --- | --- | --- | --- | --- | --- | --- | --- | --- | --- | --- | --- |
| **Cust (2016)** | Yes | Yes | Yes | Yes | Yes | No | Yes | Yes | CT | Yes | No | CT |
| **Sembi (2018)** | Yes | Yes | Yes | Yes | Yes | Yes | Yes | Yes | Yes | No | No | Yes |

Table 6.S Mixed methods studies combining RCT and qualitative methods (part 2)

| **Authors** | **5.1 Is there an adequate rationale for using a mixed methods design to address the research question?** | **5.2 Are the different components of the study effectively integrated to answer the research question?** | **5.3 Are the outputs of the integration of qualitative and quantitative components adequately interpreted?** | **5.4 Are divergences and inconsistencies between quantitative and qualitative results adequately addressed?** | **5.5**  **Do the different components of the study adhere to the quality criteria of each tradition of the methods involved?** | **Comments** |
| --- | --- | --- | --- | --- | --- | --- |
| **Cust (2016)** | Yes | No | No | No | Yes | No drop out.  Outcome data for 15/15 (100%).  Weak thematic analysis, few quotations, unattributed. |
| **Sembi (2018)** | Yes | No | No | No | Yes | 12/14 (86%) completed intervention.  Outcome data for 22/28 (79%) post-intervention, 14/28 (50%) at 6 month follow up.  2 participants recruited despite falling outside exclusion criteria.  Underpowered.  Comprehensive qualitative analysis with quotations. |
