# Supplementary material for: Community-based perinatal mental health peer support: a realist review
Source: BMC Pregnancy Childbirth. 2023 Aug 9;23:570. doi: 10.1186/s12884-023-05843-8 (PMC10410814; doi:10.1186/s12884-023-05843-8)
Supplement: Supplementary file 4 — Supplementary Material 4 [file 12884_2023_5843_MOESM4_ESM.docx]

Details of documents included in the realist review and the interventions they describe

| **Intervention #** | **Author (date), [main article reference],**  **location** | **Methodology** | **Number of qualitative informants** | **Number of participants in quantitative study** | **Type of peer support and setting** | **Frequency** | **Perinatal mental health criteria** | **Referral of mothers** | **Reported characteristics of mothers** | **Baby’s age** | **Inclusion of mothers with mental health history** | **Training for volunt-eers** | **Characteristics of volunteers** | **Co-creation by peers** | **Use in review** |
| --- | --- | --- | --- | --- | --- | --- | --- | --- | --- | --- | --- | --- | --- | --- | --- |
| 1 | **Anderson (2013) [54], USA** | Qualitative: interviews (phenomen-ology) | 7 mothers | N/A | Group  (leader unclear)  No details of setting | No details | Postnatal depression (self-defined) | By doctor or self-referral | Aged 18+ | No details | No details | N/A | N/A | No details | Limited |
| 2 | **Cust (2016) [55], UK** | Pilot RCT: psychological scores, interviews and log books | 11 mothers  7 volunteers | 30 (15 received peer support) | 1:1 face-to-face.  At home or place of mother's choice | 6 weekly sessions x 1 hour | Postnatal depression (EPDS 11-16) | Health visitor | Aged 25-35.  All White British.  All first time mothers.  All had post-secondary education. | 6 weeks | Excluded | 1 day | Recovered from mild to moderate depression. | Yes | Medium |
|  | **Carter et al (2018) [56], UK** | Qualitative: interviews and log books | 7 volunteers |  |  |  |  |  |  |  |  |  |  |  | High |
| 3 | **Carter et al (2019) [57], UK** | Qualitative (within feasibility study): interviews and log books | 9 mothers  4 volunteers | 20 (10 received peer support) | 1:1 face-to-face.  At home or place of mother's choice | 6 weekly sessions x 1 hour | Antenatal depression (Whooley screening questions) | Community midwife | All spoke English.  All first time mothers. | Pregn-ancy (28-30 weeks) | Excluded | 2 days | Recovered from antenatal depression. | Yes | Medium |
|  | **Cust and Carter (2018) [58], UK** | Researcher’s reflections on process | N/A |  |  |  |  |  |  |  |  |  |  |  | High |
| 4 | **Chen et al (2000) [59], Taiwan** | RCT: psychological scores | N/A | 60 (30 received peer support) | Group (led by nurse).  No details of setting. | 4 weekly sessions x 1.5-2 hours | Postnatal distress (Taiwanese BDI 10+) | On postnatal wards | Aged 18+  50% at least high school.  Two-thirds first time mothers.  Range of social class. | 6-10 weeks | No details | N/A | N/A | No | Limited |
| 5 | **Dennis (2003) [60], Canada** | Pilot RCT: psychological scores, questionnaires | 20 mothers & 16 volunteers | 42 (20 received peer support) | 1:1 by telephone | No fixed amount, during 2+ months. | Postnatal depression EPDS >9 | Public health nurses | Aged 18+.  All spoke English.  Most born in Canada.  Most had a partner.  Most had post-secondary education. | 8-12 weeks | Excluded (if recent or chronic) | 4 hours | Recovered from postnatal depression.  Most were married.  Most had post-secondary education. | No | Medium |
| 6 | **Dennis et al (2009) [61], Canada** | RCT: psychological scores, questionnaire | 221 mothers | 701 (349 received peer support) | 1:1 by telephone | No fixed amount,  during 12+ weeks. | Postnatal depression EPDS >9 | Public health nurses | Aged 18+.  All able to speak English.  93% married.  80% had post-secondary education.  21% non-Canadian. | 2 weeks | Excluded if currently taking medicat-ion | 4 hours | Recovered from postnatal depression.  82% married.  92% had post-secondary education.  39% first time mothers.  54% non-Canadian. | No | High |
|  | **Dennis (2010) [62], Canada** | Questionnaire | 221 mothers |  |  |  |  |  |  |  |  |  |  |  | High |
|  | **Dennis (2013) [63], Canada** | Questionnaire | 121 volunteers |  |  |  |  |  |  |  |  |  |  |  | Medium |
|  | **Dennis (2014) [64], Canada** | Description of process | N/A |  |  |  |  |  |  |  |  |  |  |  | Limited |
| 7 | **Duskin (2005) [77], USA** | Qualitative: interviews | 5 mothers | N/A | Group (led by graduate students).  At medical centre. | No details | Postnatal depression & anxiety (self-report) | No details | Aged 37-42.  4 White, 1 Latina.  All first time mothers.  All married.  All high socio-economic status. | 2 weeks – 4 months | Included | N/A | N/A | Unclear | High |
| 8 | **Eastwood et al (1995) [65], UK** | Quasi-experimental pre-test/post-test: psychological scores, questionnaire; researchers’ observations | 8 mothers | N/A | Group (led by health visitors).  At commun-ity clinic. | 12 weekly sessions. | Postnatal depression and anxiety | No details | Aged 19-35.  Social class II-V  Mostly not first time mothers.  Most had a partner. | No details | Included | N/A | N/A | No | Limited |
| 9 | **Field et al (2013a) [66], USA** | Parallel group RCT peer support vs IPT:  psychological scores, cortisol levels | N/A | 44 (22 received peer support) | Group (no leader).  No details of setting. | 12 weekly sessions x 20 minutes. | Antenatal depression (clinical interview) | At ultrasound clinic | Aged 20-38.  Mostly Hispanic or African-American.  Mostly low income, high-school education.  >1/3 had no partner. | Pregn-ancy | Excluded | N/A | N/A | No | Medium |
| 10 | **Field et al (2013b)** **[67], USA** | Parallel group RCT peer support vs yoga:  psychological scores, cortisol levels | N/A | 96 44 received peer support) | Group (no leader).  No details of setting. | 12 weekly sessions x 20 minutes. | Antenatal depression (clinical interview) | At ultrasound clinic | Aged 18-40.  Mostly Hispanic or African-American.  Mostly low income, high-school education.  >1/3 had no partner. | Pregn-ancy | Excluded | N/A | N/A | No | Medium |
| 11 | **Gjerdingen et al (2013) [68], USA** | Pilot RCT peer support vs doula or control: psychological scores | N/A | 39 (13 received peer support) | 1:1 by telephone | No fixed amount. | Postnatal depression PHQ-9 (cut off not stated) | Hospitals, local practices, websites, Early Childhood & Family Education | Mean age 29.7.  95% White.  84% married.  44% first time mothers.  74% had post-secondary education.  Mostly middle or high income. | From birth | No details | 0.5 day | Recovered from postnatal depression. | No | Limited |
| 12 | **Letourneau et al (2015) [69],**  **Canada** | Quasi-experimental pre-test/post-test: psychological scores, questionnaire | 34 mothers | 64 | 1:1 by telephone | Weekly for 4-12 weeks | Postnatal depression (EPDS 12-19) | Telehealth nurses, public health nurses | Aged 17–43.  74% spoke English, 26% spoke French.  71% had post-secondary education.  41% babies had spent time in neonatal intensive care. | Up to 24 months | Included | 1 day | Recovered from postnatal depression.  Aged 23-40.  Spoke English or French. | Yes | Limited |
|  | **Letourneau et al (2016) [70], Canada** | Description of process: qualitative interviews | 26 stakeholders | N/A |  |  |  |  |  |  |  |  |  |  | Limited |
| 13 | **Ludwick (2017) [78],**  **USA** | Description of process, researcher’s field notes | N/A | N/A | Telephone group (led by graduate student). | Weekly | Postnatal depression (self-defined) | Self-referral | No details | 3 weeks to 14 months | No details | N/A | N/A | Unclear | Limited |
| 14 | **Maley (2002) [71], USA** | Description of process | N/A | N/A | Group (led by nurse/ social worker).  No details of setting. | Monthly | Postnatal depression (self-defined) | Self-referral, doctors, community organisations | Most had a supportive partner or family. | No details | No details | N/A | N/A | Yes | Limited |
| 15 | **Montgomery et al (2012) [72], Canada** | Qualitative: observation and interviews (ethnography) | 7 mothers observed in group & 3 interviewed | N/A | Group (led by peer).  Commun-ity location accessibleby public transport. | 5 weekly sessions x 2 hours | Postnatal depression (self-defined) | Self-referral | Aged 18-30.  All spoke English.  All had at least high school education.  All had a partner. | No details | No details | N/A | N/A | Yes | Limited |
| 16 | **Pitts (1999) [73], UK** | Retrospective psychological scores & questionnaires | 34 mothers, 32 health professionals | N/A | Group (led by health visitors).  No details of setting. | Weekly | Postnatal depression (EPDS 12+, but admitted 2 women with 9) | Health visitors, GPs | No details | No details | No details | N/A | N/A | No | Medium |
| 17 | **Prevatt et al (2018) [74],**  **USA** | Quasi-experimental pre-test/post-test:  psychological scores, questionnaire with open text | 25 mothers | 45 | Group  (led by peers, with medical advisor). Waiting room of medical practice. | Weekly x 90 minutes. | Postnatal depression (self-defined) | Self-referral | Aged 22 to 45.  86% White.  85% married.  89% had post-secondary education.  58% first time mother. | No details | Included | No details | Recovered from postnatal depression. | Yes | Medium |
| 18 | **Sembi (2018)** **[79],**  **UK** | Pilot RCT & RCT: psychological scores, questionnaires, activity logs, interviews | PILOT:  6 mothers  RCT: 12 mothers  & 6 volunteers | RCT 28 (14 received peer support) | 1:1 by telephone | No fixed amount,  lasted 4 months | Postnatal depression (PILOT: EPDS 10-21 or Whooley questions; MAIN: EPDS 10-22) | Health visitor, GP, self-referral | Aged 16+.  All spoke English.  86% White British.  14% did not state ethnicity. | Up to 24 months | Included | 8 hours (4 x 2 hour sessions) | Recovered from postnatal depression.  All White British. | No | High |
| **19** | **Shorey et al (2019) [75],**  **Singapore** | RCT: psychological scores, questionnaires | N/A | 138 (69 received peer support) | 1:1 by telephone/ text/ WhatsApp | No fixed amount,  lasted 4+ weeks | Postnatal depression (EPDS >8) | Nurses on postnatal ward | Aged 23-43.  43% Chinese.  34% Malay.  Mostly first time mothers.  96% married.  60% had post-secondary education. | From birth | Excluded | 0.5 day | Recovered from postnatal depression.  Aged 21+.  All spoke English.  42% Chinese  34% Malay | No | Limited |
|  | **Shorey and Ng (2019) [76], Singapore** | Qualitative interviews | 10 mothers & 19 volunteers | N/A |  |  |  |  | Aged 25 -40. 50% Chinese.  45% Malay. |  |  |  | Recovered from postnatal depression.  Age 25-54  90% Chinese |  | Medium |
| **20** | **Acacia Family Support (2019) [80],**  **UK** | Psychological scores, questionnaires | N/A | 159 mothers | 1:1 face-to-face.  Communi-ty centre. | No fixed amount,  No time limit. | Antenatal/  postnatal depression or anxiety (self-defined) | Self-referral, midwives, health visitors, GPs, other agencies | No details for peer support specifically. For all services: 1/3 from Black, Asian or Minority Ethnic communities.  50% living in deprived areas. | Preg-nancy and up to 2 years | No details | 1 day core | Majority had experience of postnatal depression or other mental health issues. | Yes | Limited |
| **21** | **Fairbairn and Kitchener (2020) [81],**  **UK** | Psychological scores, questionnaires | N/A | 126 mothers | 1:1 face-to-face.  At home or place of mother's choice. | 6 sessions. | Antenatal/  postnatal depression or anxiety (EPDS and GAD7) | Self-referral or any health profession-al | No details. | Pregn-ancy and up to 2 years | No details | unclear | First-hand experience of perinatal mental health difficulties. | Yes | Limited |
| **22** | **Lynch (2019) [82], UK** | Qualitative interviews/ focus groups; survey | 21 mothers; 16 staff & volunteers; 28 stakeholders | N/A | Group (led by peer staff).  Communi-ty centre. | Weekly - no time limit. | Any (self-defined) | Self-referral or profession-al | All White British | Pregn-ancy and up to 2 years | No details | unclear | Experience of perinatal mental health difficulties (own or supporting family/friend). | Yes | Limited |

**Key: Psychological assessment instruments**

BDI Beck Depression Inventory

EPDS Edinburgh Postnatal Depression Scale

GAD7 Generalized Anxiety Disorder 7 item scale

HADS Hospital Anxiety and Depression Scale

PHQ9 Patient Health Questionnaire 9 item scale

Whooley questions ‘During the last month, have you often been bothered by feeling down, depressed or hopeless?’ and ‘During the last month, have you often been bothered by having little interest or pleasure in doing things?’
