# Supplementary material for: Community-based perinatal mental health peer support: a realist review
Source: BMC Pregnancy Childbirth. 2023 Aug 9;23:570. doi: 10.1186/s12884-023-05843-8 (PMC10410814; doi:10.1186/s12884-023-05843-8)
Supplement: Supplementary file 5 — Supplementary Material 5 [file 12884_2023_5843_MOESM5_ESM.docx]

**Final theoretical model section A: context-mechanism configurations where the outcome is take-up of peer support**

| **Theory #** | **Context** | | **Mechanism** | | **Studies** | **Example quotation** |
| --- | --- | --- | --- | --- | --- | --- |
|  | **Social level** | **Individual level** | **Resources provided by peer support programme** | **Reasoning or reaction leading to use of peer support** |  |  |
| **1** | Cultural narratives of idealised motherhood    Stigma of mental illness | Negative self-labelling as a uniquely abnormal 'bad' mother | Offer of peer support | Mother believes peers will be empathetically understanding and trustworthy | Duskin (2005)  Sembi (2018) | *C: “I thought I was the only person in the world who must be feeling like this about my baby… I felt so guilty and I felt like I didn’t dare say anything to anybody about the way I was feeling, because I thought people would think I’m a terrible person.”*(Sembi, 2018) |
| **2** | Cultural narratives of idealised motherhood  Stigma of mental illness | Hides feelings from partner, family & friends & can't meet needs for authenticity in relationships, or lack of social network | Offer of peer support | Mother feels safe to attend because peers are outside normal social circle | Cust & Carter (2018)  Duskin 2005)  Sembi (2018) | *C: “It was also about feeling like I should be giving my friends the impression that I felt great, everything was great...and I couldn’t even summon up enough energy to carry a conversation with them.”* (Duskin, 2005)  *C-M: “You don't want to shock people by revealing some of your feelings and thought processes, it’s very difficult. You couldn’t perhaps confide in your husband as you would be able to someone on the phone.”* (Sembi, 2018)  COUNTER: “The women [who turned down peer support] also felt that they may be uncomfortable talking to a stranger, and that they were not sure if it would really offer any benefit to them.” (Cust & Carter, 2018) |
| **3** | Cultural narratives of idealised motherhood  Stigma of mental illness  Expectation that new mothers will meet social support needs through other new parents | **C-I3** Mother lacks a social network  **C-I4** Avoids new parent groups as these make her feel worse | Offer of peer support | Mother feels safe to attend because believes peers will be non-judgementally accepting | Anderson (2013)  Duskin (2005) | *C: “I hated regular mother’s groups because I hear just how well they are doing … Why am I so different than these other women? Why am I having such a hard time? What is wrong with me?”* (Duskin, 2005)  COUNTER: *“’[The generic group] was sort of like fun and social. It was something to do while we’re on maternity leave and a social outlet’…*After she disclosed her PPD experience, other group members began disclosing their own struggles with PPD.” (Anderson, 2013) |
| **4** | Primary health professionals have limited training on perinatal mental health difficulties and limited time | Conceals symptoms from professionals – fear of judgment, consequences, lack of understanding / empathy; OR DISAPPOINTING PREVIOUS EXPERIENCE OF PROFESSIONAL SUPPORT | Peer support is available by self-referral | Mother believes peer support is a safe or better alternative, trusts lived experience over professional knowledge | Carter et al. (2019)  Cust (2016)  Eastwood et al. (1995)  Lynch (2019)  Sembi (2018) | C: “They said that a lot of good advice is given from a negative viewpoint by health visitors, which undermines what little confidence they have and either causes or emphasises guilt, and this makes them reluctant to share concerns.” (Eastwood, 1995)  *C: “You’re not a hundred percent honest with official people… for fear of what might happen to your family … You [need to] have built up a level of trust first and you haven’t got time to do that with a GP, you’ve got ten minutes.”*(Sembi, 2018)  *C-M: “I find it difficult to talk to people who haven’t been through things themselves … [With my counsellor I asked] have you ever had any problems ... no? I was like, you’re learning this out of a book then. How can you tell me that this is normal what I’m feeling if you’ve never felt it yourself?”* (Sembi, 2018) |
| **5** | Primary health professionals have limited training on perinatal mental health difficulties and limited time | Mother trusts health professionals | Programme has good relationship with local health professionals and a simple referral process | Mother is referred by a health professional she trusts OR RECRUITED IN HOSPITAL | Acacia (2019)  Carter et al. (2019),  Chen et al. (2000)  Cust & Carter (2018)  Dennis et al. (2009)  Letourneau et al. (2015, 2016)  Lynch (2019)  Pitts (1999)  Sembi (2018)  Shorey & Ng (2019) | M: “Acacia’s longevity and excellent reputation can in part be attributed to our active partnership work, which has helped to embed our services across local perinatal mental health pathways. We are active participants in all of the Birmingham-based groups and networks for perinatal mental health.” (Acacia, 2019)  C: “We sought feedback from the community midwives as to why recruitment was difficult: ‘*We simply do not have time to take on this additional role.’*” (Cust, 2018) |
| **6** | Different conceptions of mental health difficulties and appropriate response | Personal conception of cause and meaning of perinatal mental health difficulties | Programme terminology matches mother's own understanding of her mental health | Mother believes that the peer support is aimed at people like her | Duskin (2005)  Sembi (2018) | *C: “Even back then, I would not have thought I was depressed, I just thought, I’m having a tough time.”* (Duskin, 2005)  *C: “When I initially went to my GP I felt very apologetic that I was wasting time, because it’s not a visible illness or anything.”* (Sembi, 2018) |
| **7** | Public health campaigns promote message ‘it's good to talk’, but there are differences in acceptability of talking to outsiders | Mother believes it is useful and acceptable to talk about mental health difficulties | Offer of peer support | Mother wants to talk about her mental health | Shorey & Ng (2019) | C: “However, mothers [in Singapore] are often dissatisfied with social support, especially due to the lack of emotional support received. This can be attributed to the conservative nature of Asian societies in which direct emotional expressions are often discouraged …This highlights an unspoken need for more emotional support for Asian mothers. Mothers often mentioned a need for a close, nonjudgmental confidante to initiate support and empathize with them.” (Shorey & Ng, 2019) |
| **8** |  | Mother has preference for cultural homogeneity or heterogeneity | Programme is able to offer a choice in cultural matching or otherwise | Mother feels safe to attend because similarity or difference matches her needs | Shorey & Ng (2019) | C-M: “In order to increase relevance to self, mothers generally preferred to be matched with a volunteer of similar age, same ethnicity, employment status, marital status, recency of childbirth, and similar ages of children.” (Shorey & Ng, 2019) |
| **9** | Limited access to perinatal mental health support, including long waiting lists | Mother wants counselling | Programme positions self as alternative for those who do not meet criteria for professional support | Mother may go to peer support as a holding position while waiting for counselling or hoping she will receive counselling-type support | Carter et al. (2019)  Eastwood et al. (1995)  Letourneau et al. (2015) | *C: “There doesn’t appear to be a lot around unless you are actually feeling completely suicidal.”* (Carter et al., 2019)  COUNTER: “[Mothers] perceived peer-support to be a different type of support from professional support and, as such, should sit alongside professional support, but not replace it.” (Sembi, 2018) |
| **10** | Social norm that mother is primarily responsible for meeting baby's needs alongside domestic responsibilities and other work | Mother has resources of time and/or money to invest in meeting her own needs | Programme offers support requiring less commitment e.g. by phone or drop in group OR PROVISION OF CRECHE / TRANSPORT, OR AT ACCESSIBLE LOCATION/AT HOME; PROGRAMME PAYS MOTHERS TO ATTEND | Mother can use peer support in ways that do not exceed her resources | Cust (2016)  Carter et al. (2019)  Dennis (2010)  Eastwood et al. (1995)  Fairbairn & Kitchener, (2020)  Field et al. (2013 a/b)  Letourneau et al. (2016)  Lynch (2019)  Prevatt et al. (2018) | M: *“I think when we’re feeling very low energy and sad, it’s very hard to force ourselves out of the home. So the phone could be your best friend, or could be the best way of getting that support.”* (Letourneau et al. 2016)  COUNTER: *“It would have been nice to talk some more [on the phone], but it is very hard to ﬁnd the time with a new baby.*’’ (Dennis, 2010) |
| **11** |  | Low social confidence | ~~Programme offers choice of 1:1 or group support~~ | Mother feels safe talking to a peer supporter 1:1 although would not feel safe in peer support group~~, or vice versa~~ | Dennis (2010)  Fairbairn & Kitchener (2020)  Pitts (1999)  Sembi (2018) | *C-M: "Sometimes a one-to-one situation could have helped as there were some things I felt I could never say to complete strangers."* (Pitts,1999) |
| **12** | TELEPHONE WIDELY USED BY TARGET COMMUNITY TO ACCESS HEALTH SERVICES | MOTHER SEES TELEPHONE SUPPORT AS CONVENIENT AND/OR PREFERS ANONYMITY | PROGRAMME OFFERS TELEPHONE SUPPORT | MOTHER IS COMFORTABLE USING TELEPHONE FOR SUPPORT | Dennis (2010)  Letourneau et al. (2016)  Sembi (2018)  Shorey & Ng (2019) | *C-M: “I just found myself telling her things that I wouldn’t tell other people …because there’s no face there, there’s no actual person there. It does feel really private.”* (Sembi, 2018)  COUNTER: *“I didn’t feel I could talk over the phone to someone I didn’t know well.”* (Sembi, 2018)  COUNTER: “Mothers and peer volunteers were highly in favor of at least one session of face-to-face meet-up, which would allow easier rapport building.” (Shorey & Ng 2019) |
| **13** | Pool of potential peer support volunteers exists in community |  | There is a peer supporter available to start support and to continue it reliably when the mother needs it. | Mother can use peer support when she needs it. | Cust (2016)  Cust & Carter (2018)  Dennis (2010)  Sembi (2018) | M: “As the recruiters, we need to provide further specification as to how time consuming the [volunteer] role may potentially be.” (Cust & Carter 2018)  M: “*I’ve not been available myself and she’s been struggling with her family to be able to call when it was appropriate for both of us.”* (Sembi, 2018) |

Key

**Normal font**: Programme theories which formed the initial theoretical model and were also present in the final model.

**CAPITALISED FONT**: Theories which were not in the initial theoretical model, but were added to the final theoretical model in the light of C-M-O analysis.

**~~Strikethrough~~:** Theories which were in the initial theoretical model, but for which no evidence was found.
