# Supplementary material for: Community-based perinatal mental health peer support: a realist review
Source: BMC Pregnancy Childbirth. 2023 Aug 9;23:570. doi: 10.1186/s12884-023-05843-8 (PMC10410814; doi:10.1186/s12884-023-05843-8)
Supplement: Supplementary file 6 — Supplementary Material 6 [file 12884_2023_5843_MOESM6_ESM.docx]

Final theoretical model section B: positive C-M-Os for mothers using peer support

| **Theory #** | **Context**  **‘*in what circumstances?’ and ‘for whom?’*** | **Positive mechanisms**  ***‘what is it that works?’ and ‘why?’*** | | **Outcomes**  ***‘does it work?’ and ‘in what respects?’*** | **Studies** | **Example quotations** |
| --- | --- | --- | --- | --- | --- | --- |
|  |  | **What happens during peer support** | **Reasoning or reaction of mother** |  |  |  |
| **14** | Cultural narratives of idealised motherhood  Stigma of mental illness  Expectation that new mothers will meet social support needs through other new parents  Negative self-labelling as a uniquely abnormal 'bad' mother  Hides feelings from partner, family & friends & can't meet needs for authenticity in relationships, or lack of social network  Mother lacks a social network  Avoids new parent groups  Cultural narratives of idealised motherhood  Stigma of mental illness  Expectation that new mothers will meet social support needs through other new parents  Negative self-labelling as a uniquely abnormal 'bad' mother  Hides feelings from partner, family & friends & can't meet needs for authenticity in relationships, or lack of social network  Mother lacks a social network  Avoids new parent groups | **Mother talks honestly and is listened to empathetically** | Feeling understood | Emotional release: able to share true self | Anderson (2013), Carter et al. (2019)  Chen et al. (2000)  Cust (2016), Dennis (2010), Duskin (2005), Eastwood et al. (1995)  Montgomery et al. (2012), Pitts (1995)  Prevatt et al. (2018)  Sembi (2018) | *M-O: "Being able to cry and laugh with a group of very understanding women was a great release."* (Pitts, 1999)  *C-M-O: “It was a release… I’d be able to go and moan to and get everything off my chest …I feel like you have to put on a brave face, like with my husband and my family and pretend everything is fine.”* (Sembi, 2018) |
| **15** |  | **Mother talks honestly and is listened to non-judgementally** | Feeling accepted by others, leading to self-acceptance | Improved self-esteem / self-concept  ~~Mother gains confidence to disclose to partner/family/friends and authentic relationships are restored~~  Mother gains confidence to attend 'normal' new parent groups | Carter et al. (2019)  Dennis (2003, 2010)  Duskin (2005)  Pitts (1999)  Prevatt et al. (2018),  Sembi (2018) Shorey & Ng (2019) | *M-O: “I told you the scariest, worst, horrible, darkest, deep feelings that I felt, and it was like nobody batted an eye. And not only did nobody bat an eye, I got loving, patient, warm, understanding…* *It just made me feel like, wow, I am a normal person.”* (Duskin, 2005)  M-O: “Some expressed that, with encouragement from the group, they started to attend mother and toddler groups, where they have made further friendships." (Pitts, 1999) |
| **16** |  | **Peers give positive feedback about her feelings and actions** | Feeling affirmed | Improved self-esteem / self-concept | Dennis (2003, 2010)  Duskin (2005)  Sembi (2018) | *M-O: “I got validation that it was okay to feel depressed.”* (Duskin, 2005)  M: “Told me that I did something well …Agree or strongly agree, 72.9%.” (Dennis, 2010) |
| **17** |  | **Peers talk about their own perinatal mental health and parenting challenges** | Mental health and parenting challenges are normalised  Compares herself to peers who are now well  Compares herself to peers who are less well  Compassion for others promotes self-compassion | Improved self-esteem / self-concept  Hope for recovery  Sense of perspective | Anderson (2013)  Chen et al. (2000)  Cust (2016)  Dennis (2010)  Duskin (2005)  Montgomery et al. (2012)  Pitts (1999)  Prevatt et al. (2018),  Sembi (2018) | *C-M-O: "It was the most helpful part of my recovery. I spent most of my time believing I was useless and not a very good mother for not being ecstatic about the birth of my child, talking to all of the others made me realise I was normal."* (Pitts, 1999)  *M-O: "When I first attended it was nice to see women who were already getting better so I knew there was light at the end of the tunnel."* (Pitts, 1999)  *M-O: “I was like, wow, she’s feeling exactly the way that I felt, so I have done some progress because I don’t feel that way anymore.”* (Duskin, 2005)  C-M-O: “When the mothers felt judgmental and pessimistic about there [sic] own situations, through social comparison they were able to view a woman in a similar situation with compassion and objectivity and then to alter their negative view of themselves.” (Duskin 2005) |
| **18** | MOTHER TRUSTS PERSONAL EXPERIENCE OVER PROFESSIONAL KNOWLEDGE | **Peers share ideas about self-care, coping with perinatal mental health, parenting, medication, mental health services, other community services** | Mother gains information she finds credible and encouragement to try new things | Increased coping strategies and lower stress  Increased take-up of mental health services  Increased use of other community services | Chen et al. (2000)  Cust (2016)  Dennis (2003/2010)  Duskin (2005)  Fairbairn & Kitchener (2020)  Montgomery et al. (2012)  Pitts (1999)  Prevatt et al. (2018)  Sembi (2018) | M-O: “Women identiﬁed individual mothering challenges and solicited remediating information… The group created a repertoire of practical mothering strategies.” (Montgomery et al., 2012)  C-M: “The mothers viewed their [peer supporter] as 'an expert' – [she] had experienced what they were currently experiencing and 'had survived' - therefore any guidance and advice that they could offer 'was tried and tested.'” (Cust, 2016)  O: “Attendees responded that the program prompted the initiation of treatment.” (Prevatt et al., 2018) |
| **19** | Limited access to perinatal mental health support, including long waiting lists | **Peers use therapeutic techniques such as ~~reframing, challenging negative cognitions,~~ ENCOURAGING SELF-CARE** | Mother experiences therapy-lite  Mother gains access to techniques she can try | Increased coping with perinatal mental health difficulties | Sembi (2018) | *M-O: “She was really good at suggesting I go and do something really nice for myself. Just to remind myself to do little things for me and to actually set a timescale.”* (Sembi, 2018)  COUNTER: “The [volunteers] simply wanted to provide support to the women as a fellow ‘mother to mother’.” (Cust & Carter, 2018) |
| **20** | Mother is sufficiently well to give as well as receive peer support | **Opportunity for reciprocal support** | Mother offers support to other mothers in a group | Self-esteem Finding meaning in own experiences | Anderson (2013) Duskin (2005)  Montgomery et al. (2012) | *C-M: “Knowing that I was doing better and being able to share that with people, was important from the point of view of being able to reach out to them the way people had reached out to me.”* (Duskin, 2005) |
| **21** | Mother has low self-esteem | **Programme offers support from volunteers who are not paid for their time** | Mother experiences herself as worth another person’s time | Self-esteem  Feels cared about | Acacia (2019) Shorey & Ng (2019) | *M-O: “The main thing I remember was feeling that finally someone cared, [peer supporter] … showed that she and others cared whether I was here or not.”* (Acacia, 2019)  COUNTER: *“I just felt like I was an extra burden for [the volunteer] … she was busy with her own life.”* (Sembi, 2018) |
| **22** | Mother lacks a social network | **Same peers attend group over time, or 1:1 peer supporter is well matched**  ~~Peer support is for mothers whose mental health difficulties are specifically connected to having a baby, and are mild-to –moderate~~  MOTHERS AT GROUP ARE SOCIO-DEMOGRAPHIC-ALLY SIMILAR | Mother forms meaningful relationships (that may continue outside group) | Reduced loneliness Increased social network | Dennis (2003/2010)  Duskin (2005)  Lynch (2019)  Pitts (1999)  Prevatt et al. (2018)  Sembi (2018)  Shorey & Ng (2019) | *C-M-O: “A sense of belonging that I had lost because I had isolated myself from my old friends.”* (Duskin, 2005)  M-O: “The women described the positive impact of accessing support, warmth and meaningful friendships with women who had/have shared experiences as transformative.” (Lynch, 2019)  COUNTER*: “Sometimes, I don’t feel very connected to the person that I call...so, sometimes, it gets awkward during the phone conversation”* (Shorey & Ng, 2019) |
| **23** | Social norm that mother is primarily responsible for meeting baby's needs alongside domestic responsibilities and other work  MOTHER FEELS TRAPPED AT HOME WITH BABY | PEER SUPPORT SESSIONS ARE HELD REGULARLY | PEER SUPPORT IS A REASON TO LEAVE THE HOUSE | MOTHER GAINS STRUCTURE TO HER WEEK  SENSE OF ACHIEVEMENT IN MANAGING TO ATTEND | Duskin (2005)  Pitts (1999) | *C-M-O: "When you are depressed it is very hard to get organised and you tend just to stay at home feeling worse. This group made me get organised and gave me something to look forward to!”* (Pitts, 1999) |
| **24** |  | GROUP PROVIDES CHILDCARE OR HELD AT TIME WHERE MOTHER GOES ALONE | PEER SUPPORT REPRESENTS A BREAK FROM CHILDCARE | MOTHER HAS TIME FOR HERSELF | Lynch (2019)  Pitts (1999) | C-M-O: “The crèche is not viewed as a ‘bolt on’ or ‘nice to have’ by most service users but rather as crucial to the successful delivery of outcomes. For many women the time they use crèche is the only time they take away from their children and this time spent on self-care (even if it’s not very much) is essential to their improved wellbeing.” (Lynch, 2019) |
|  | ? | 1:1 support  Group support | ? | Reduction in self-report depression scores at endpoint | Chen et al. (2000)  Cust (2016), Dennis (2003), Dennis et al. (2009), Field et al. (2013 a/b)  Gjerdingen (2013)  Sembi (2018)  Shorey et al. (2019) | RCT evidence:  Statistically significant reduction in symptoms of postnatal depression after one-to-one peer support for 12 weeks (Dennis, 2009) or 3 months after birth (Shorey et al. 2019).  Statistically significant reduction in symptoms of postnatal depression after 4 weeks of group peer support (Chen et al., 2000). |
|  | ? | 1:1 support  Group support | **?** | Non-significant reduction in self-report anxiety scores at endpoint | Dennis et al. (2009)  Field et al. (2013 a/b)  Shorey et al. 2019  COUNTER: Eastwood et al. (1995) | RCT evidence: Non-significant trend in reduced symptoms of anxiety favouring the intervention arm after one-to-one peer support for 12 weeks (Dennis, 2009) or 3 months after birth (Shorey et al. 2019).  COUNTER: Pre-test/post-test evidence: Anxiety scores unchanged or increased after group peer support for 12 weeks (Eastwood et al. 1995) |

Key

**Normal font**: Programme theories which formed the initial theoretical model and were also present in the final model.

**CAPITALISED FONT**: Theories which were not in the initial theoretical model, but were added to the final theoretical model in the light of C-M-O analysis.

**~~Strikethrough~~:** Theories which were in the initial theoretical model, but for which no evidence was found.
