# Supplementary material for: Community-based perinatal mental health peer support: a realist review
Source: BMC Pregnancy Childbirth. 2023 Aug 9;23:570. doi: 10.1186/s12884-023-05843-8 (PMC10410814; doi:10.1186/s12884-023-05843-8)
Supplement: Supplementary file 7 — Supplementary Material 7 [file 12884_2023_5843_MOESM7_ESM.docx]

Final theoretical model section C: negative C-M-Os for mothers using peer support

| **Theory #** | **Context** | **Negative mechanisms** | | **Negative outcomes** | **Studies** | **Example quotations** |
| --- | --- | --- | --- | --- | --- | --- |
|  |  | **What happens during peer support** | **Reasoning or reaction of mother** |  |  |  |
| **N1** | Low self-esteem and low internal locus of control | **Peers talk about their own perinatal mental health and parenting challenges but do not validate others’ experiences and feelings**  **Programme has inclusive approach and the mothers are not similar in background or mental health experience** | Mother feels unvalidated, abnormal. | Increased sense of abnormality and shame  Loss of authenticity | Carter et al. (2018)  Dennis (2010) | M-O: “While blatantly negative effects were rare in this trial, almost 10% of mothers reported that the peer minimized their problems.” (Dennis, 2010)  M: “[The volunteer] confessed that although she did want to provide all of the right support to her allocated mother, she also wanted to: *take control and tidy the mother and her house up.*” (Carter, 2018) |
| **N2** | Low self-esteem and low internal locus of control | **Peers talk about their own perinatal mental health and parenting challenges** | Mother feels bad about not coping when others are worse, or fears getting worse | Self-criticism / reduced emotional wellbeing | Cust & Carter (2018)  Duskin (2005)  Pitts (1999)  Prevatt et al. (2018) | *M-O: “She made me really anxious because it didn’t seem like she was getting any better … back to my old fear, am I never going to get better?”* (Duskin, 2005)  *M-O: “(H)earing about things that all of the other moms worried about made me more anxious. That’s why I personally only attended one meeting.”* (Prevatt, 2018) |
| **N3** |  | **Peers talk about their own perinatal mental health and parenting challenges** | Mother feels discouraged that others are getting well more quickly | Self-criticism / reduced emotional wellbeing | Dennis (2010)  Duskin (2005) | *M: ‘‘[The peer supporter] made it seem that her life was much better than mine.’’* (Dennis, 2010) |
| **N4** |  | **Peers focus on sharing negative feelings and experiences, peers do not use therapeutic techniques**  **PEERS DO NOT COMFORT EACH OTHER WHEN DISTRESSED**  **Group does not have effective facilitation or peer supporters are not well selected/ trained** | Mother responds to others’ negativity or distress with own negativity and distress | Reduced emotional wellbeing, anxiety over own recovery and sadness over others’ suffering | Carter et al. (2018)  Pitts (1999)  Prevatt et al. (2018) | M-O: “Q: What could have helped you more? A: *Always having a group discussion at the end on a happy subject, letting others input ideas/ suggestions about how other people in the group could make their lives/feelings better."* (Pitts, 1999)  *M-O: "When they became upset, I could see they wanted to be comforted, but no-one seemed to do that. That made me very uncomfortable."* (Pitts, 1999) |
| **N5** |  | **PEERS ARE JUDGEMENTAL OR DIRECTIVE.**  **GROUP DOES NOT HAVE EFFECTIVE FACILITATION OR PEER SUPPORTERS ARE NOT WELL SELECTED/ TRAINED** | MOTHER FEELS JUDGED AND DISEMPOWERED | REDUCED SELF-ESTEEM, DISEMPOWERMENT | Carter et al. (2018)  Cust (2016)  Duskin (2005) | M: “She admits she was initially judgmental of other mothers she saw who were taking care of themselves, and believed this meant they were not good mothers.” (Duskin, 2005)  M: “Each [peer supporter] wanted to attempt to find their own possible solution to their mother’s problem.” (Cust, 2016) |
| **N6** |  | **THERE IS NOT TIME TO SPEAK OR MOTHER IS INTERRUPTED (GROUP DOES NOT HAVE EFFECTIVE FACILITATION OR THERE IS NO LIMIT ON NUMBERS)** | MOTHER FEELS SHE IS NOT LISTENED TO | FRUSTRATION, NOT FEELING HEARD | Pitts (1999)  Prevatt et al. (2018) | M: “Sometimes moms talked over each other, especially when groups got over 5 + people.” (Prevatt, 2018) |
| **N7** | Low social confidence | **MOTHER FEELS PRESSURE TO SPEAK TO PEER SUPPORTER** | PEER SUPPORT BECOMES AN ADDITIONAL STRESSFUL SOCIAL RELATIONSHIP | STRESS | Sembi (2018) | *M-O: “It felt more pressured because I felt like I just kind of had to talk to [peer-supporter] though I didn’t feel like I wanted to.”* (Sembi, 2018) |
| **N8** | Low social confidence | **Peer support becomes a safe bubble but mother is unable to move beyond it to authentic relationships with non-peers** | Mother is distressed about ending of support that she relies on. | Anxiety about ending.  Loss of social support.  ~~Grief and sense of loss after ending.~~ | Carter et al. (2018/2019)  Cust (2016)  Eastwood et al. (1995) | *M-O: “I dreaded my visits ending, I was really going to miss this support.”* (Carter, 2019) |
| **N9** | ~~Low social confidence~~ | **~~Attempts at friendship unsuccessful~~** | ~~Reinforces sense of social failure~~ | ~~Reduced social confidence and self-esteem~~ |  |  |
| **N10** |  | **~~Peers share unhelpful ideas about self-care, coping with perinatal mental health, parenting, medication, mental health services~~**  **~~Group does not have effective facilitation~~** | ~~Mother is influenced by poor advice from group members, not redressed by facilitator~~ | ~~Lose confidence in mental health services, use ineffective or harmful coping strategies~~ |  |  |

Key

**Normal font**: Programme theories which formed the initial theoretical model and were also present in the final model.

**CAPITALISED FONT**: Theories which were not in the initial theoretical model, but were added to the final theoretical model in the light of C-M-O analysis.

**~~Strikethrough~~:** Theories which were in the initial theoretical model, but for which no evidence was found.
